# Long-Term Economic Sustainability of Humanitarian Logistics—A Multi-Level and Time-Series Data Envelopment Analysis

**DOI:** 10.3390/ijerph18052219

**Published:** 2021-02-24

**Authors:** Matthias Klumpp, Dominic Loske

**Affiliations:** 1 Fraunhofer Institute for Material Flow and Logistics IML, 44227 Dortmund, Germany; 2 Institute for Logistics and Service Management, FOM University of Applied Sciences, 45130 Essen, Germany; dominic.loske@fom-net.de; 3 Department of Business Administration, Georg-August-University of Göttingen, 37073 Göttingen, Germany; 4 Faculty of Business and Law, UCAM Universidad Católica San Antonio de Murcia, 30107 Guadalupe, Spain

**Keywords:** DEA, window analysis, multi-level efficiency, humanitarian logistics efficiency

## Abstract

Although resources are scarce and outputs incorporate the potential to save human lives, efficiency measurement endeavors with data envelopment analysis (DEA) methods are not yet commonplace in the research and practice of non-government organizations (NGO) and states involved in humanitarian logistics. We present a boot-strapped DEA window analysis and Malmquist index application as a methodological state of the art for a multi-input and multi-output efficiency analysis and discuss specific adaptions to typical core challenges in humanitarian logistics. A characteristic feature of humanitarian operations is the fact that a multitude of organizations are involved on at least two levels, national and supra-national, as well as in two sectors, private NGO and government agencies. This is modeled and implemented in an international empirical analysis: First, a comprehensive dataset from the 34 least developed countries in Africa from 2002 to 2015 is applied for the first time in such a DEA Malmquist index efficiency analysis setting regarding the national state actor level. Second, an analysis of different sections in a Rohingya refugee camp in Bangladesh is analyzed based on a bootstrapped DEA with window analysis application for 2017, 2018, and 2019 quarter data regarding the private NGO level of operations in humanitarian logistics.

## 1. Introduction

Humanitarian logistics is an important field of general interest and research connected to the domain of public health [1,2,3]. As Doerner, Gutjahr, and van Wassenhove already described in 2011, specifically operations research (OR) interest in humanitarian logistics has picked up since about 15 years ago due to major globally recognized disasters at this time and the awareness of worldwide interconnectedness of risks [4]. This has been prolonged since then as global interrelations keep growing and are accompanied by increased means of OR methods as well as logistics and operations capabilities [5,6,7]. However, in most cases, OR methods especially addressing efficiency are applied at the “fringes” of humanitarian logistics [8,9,10,11], e.g., the cross-sectors toward civil protection or health care systems. For example, Li, Zhu, and Zhuang outline an efficient data envelopment analysis (DEA) regarding the fire protection service in the US [12], or Savaser et al. describe a transportation problem and optimization for organ transplants in Turkey [13]. Few OR publications directly address humanitarian operations such as e.g., Noyan and Kahvecioglu, presenting a supply chain management (SCM) branch-and-cut algorithm for last mile distribution in disaster situations [14]. As with these examples, there are no specific contributions regarding efficiency analysis for the strategic level of humanitarian operations, e.g., with the data envelopment method (DEA) proposed by Charnes, Cooper, and Rhodes [15] based on the works of Koopmanns [16] and Farrell [17].

The DEA method was extended e.g., by Banker, Charnes, and Cooper for variable returns to scale settings [18] or Färe and Grosskopf with a network setup [19] and is today one of the most applied efficiency calculation techniques [20]. This specific gap of missing efficiency research in humanitarian operations is exemplified also by Abidi et al. regarding the general question of performance management in humanitarian operations [21]. This is despite the fact that the joint aim of all research and management endeavors should be the optimization and overall resource efficiency specifically in humanitarian operations [22,23,24,25]—it can be stated that it is an important aim to improve our help in times of need such as e.g., in disaster and refugee situations. The lack of efficiency analysis endeavors is connected to the specific data-gathering problem in humanitarian operations due to the characteristics of this field in itself: Humanitarian operations are characterized by specific settings in comparison to other industry and logistics operations in for-profit supply chains. For example, such characteristics provide specific challenges for standard operations research methods in the field of efficiency analysis—although a measurement of efficiencies can be associated with a multitude of potential advantages.

First, the diversity of actors allows for an assumption of very diverse efficiency levels when comparing such actors in humanitarian logistics—enabling potential efficiency improvements by benchmarking and other comparative management methods. Second, especially funders for humanitarian operations such as public and private donors demand a specific and detailed analysis or reporting of the resources spent. Reporting efficiency values in addition to output performance only provide an additional benefit to actors and their funding partners. Third, the global and often short-term nature of humanitarian operations defines also a specific challenge to arrive at high efficiency levels; therefore, every additional information and hint toward improvement potential is highly welcome. Fourth, improved efficiency would possibly enable a transmission in very important output increases, especially human lives saved in disaster or dire development situations. The specifics of humanitarian operations first and foremost include the question of a multitude of actors on the national and supra-national level as well as in the government and non-government (private/non-profit) sector. Such specific settings as depicted hitherto in the following table have to be incorporated in an adequate efficiency measurement in order to provide reliable efficiency measures and therefore resource allocation and management decision options (Table 1). Global private non-profit actors, as well as national government actors, are addressed in the two presented DEA models within this paper (*Italics*).

Therefore, this paper addresses the research gap of an in-depth efficiency analysis with the data envelopment method for the specific characteristics of humanitarian operations such as the question of multiple actor levels and time series requirements between inputs and outputs during humanitarian relief and development operations. The specific contribution of this paper is presented by the fact that (i) specific OR challenges regarding an efficiency analysis in humanitarian operations are defined and (ii) a remedy is proposed with two specific adapted state-of-the-art bootstrapped DEA models for the national as well as non-government organizations (NGO) operations level; from that, (iii) a further method extension toward regression analysis and prognosis is outlined to further the OR result impact for real-life management decisions in humanitarian operations.

The paper is structured as follows: Section 2 outlines existing literature contributions from OR as well as specific challenges and data availability in humanitarian settings on a global scale. The third section develops two specified DEA analysis models for humanitarian operations. Section 4 outlines results obtained with this DEA setting, and Section 5 describes the discussion for these results. Section 6 outlines conclusions and an outlook. 

## 2. Literature Review

Regarding existing research work in the field of efficiency analysis for humanitarian operations, it is relevant to recognize that distinct operations research work in this sector has been applied e.g., with journal special issues such as OR Spectrum in 2011 [11] or the European Journal of Operational Research in 2018 [26]. In addition, many research publications regarding OR applications in humanitarian operations are scattered among different journals and topical areas. For example, a bordering area is the question of health care and civil security protection such as e.g., emergency and fire protection services and networks. This is outlined in the following table and shows vividly that the specific area of efficiency analysis addressed here has scarcely applied to humanitarian operations, and it has not been applied at all using the DEA technique (see Table 2).

In the field of humanitarian operations, in many cases, operations research concepts and techniques are applied: For example, Acimovic and Goentzel use models to assess the humanitarian response capacity [33]. Roh, Shin, and Seo (2018) discuss warehouse location topics for humanitarian operations [34], and Carlanda, Goentzel, and Montibeller (2018) present agent models for multi-echelon humanitarian operations [41]. Regarding specifically efficiency analysis with the DEA technique for health care and civil security are applied by e.g., Ustün (2016) evaluation the disaster resilience capacity in Turkey with the DEA technique [42]. Nahangi, Chen, and McCabe (2019) use DEA for a safety and efficiency evaluation in construction [44]. For example, Barakac and Dahooei (2018) apply DEA in a safety evaluation for airlines [51]. For example, for health care and civil security applications, Decerle et al. (2019) outline an ant colony optimization for the field of home health care operations [27]. Grenouilleau et al. (2019) use a set partitioning heuristic for health care routing and scheduling [28]. Ni et al. (2019) apply a deep belief network and principal component analysis in order to evaluate and improve civil aviation safety [32].

Regarding the specific question of efficiency analysis in humanitarian operations, only Jola-Sanchez et al. (2016) can be identified, identifying total factor productivity regarding hospital capacities in humanitarian operations [52]. However, for applications of the DEA technique, no specific publications are visible in the relevant journals and search instruments. This constitutes the major research gap this paper is addressing.

The research gap can partly be explained by this fact: Data evaluation and gathering for efficiency analysis such as with the DEA technique is hard due to several specifics in the strategic and operational setup of humanitarian operations. These are listed as follows:

- The time factor is critical and seldom forewarnings are accurate, making pre-planning hard or impossible;
- In most cases, there are multiple organizations involved in humanitarian operations from different sectors, fields, and countries;
- Multiple levels are included e.g., supra-national, national, and regional, from the state and private sector;
- In many cases, incomplete documentation technology can be found, so electronic data are seldom collected on operations;
- Finally, there are differing definitions and data standards regarding what sort of data to collect.

Nevertheless, data gathering is seen as one of the crucial elements of successful humanitarian operations research besides model building, including the complexity and short-term nature of such operations e.g., by Kovacs and Moshtari (2019) [53], and for example, the United Nations Office for the Coordination of Humanitarian Affairs (UN OCHA) is trying to remedy this on an international level [54]. Therefore, the next section is paying special attention to a DEA model development in relation to available and sensible data for humanitarian operations.

## 3. Methods: DEA Specification for Humanitarian Operations

### 3.1. National Governments and Agencies

**Figure 1:**
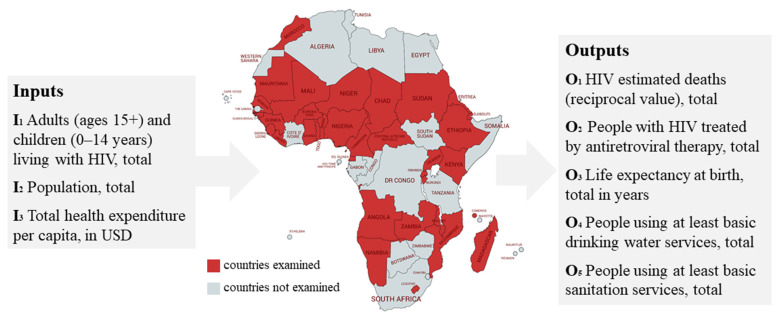
Data envelopment analysis (DEA) model for national governments and agencies.

A DEA method specification is developed in this section in relation to available and sensible data addressing humanitarian operations on a national actor level. First, a time series report for health nutrition and population statistics published by the World Bank was used [55]. The database provides key health, nutrition and population statistics gathered from a variety of international and national sources. Themes include global surgery, health financing, HIV, immunization, infectious diseases, medical resources and usage, noncommunicable diseases, nutrition, population dynamics, reproductive health, universal health coverage, and water and sanitation. The database has a temporal coverage from 1960 to 2018, is quarterly updated, and contains 104,896 datasets. The evaluation of humanitarian operations concentrated on 34 African countries. It is assumed that efficiency evaluation and the use of this information for management measures has the highest impact and therefore is a requirement in less developed countries. This touches one of the global fairness principles in humanitarian logistics as usually the less capable countries and communities are affected the most by natural disasters and for example have higher risks for climate change risks than other countries. Therefore, the analysis concentrates on the countries “most in need”. The selection of these countries is based on a downward-sorted list of the Human Development Index (HDI), whereby a few states were excluded due to a lack of available data. As infections, treatments, and deaths of the Human Immunodeficiency Viruses (HIV) are core challenges in African countries, the DEA model uses the total amount of adults (ages 15+) and children (0–14 years) living with HIV per country as an input (I_1_). It is supplemented by the total population (I_2_), aspiring to take the tax volume and the degree of development for public facilities into account. From a monetary value point of view, the current health expenditure per capita in $ USD was integrated to quantify national investment strength. Furthermore, external health expenditures per capita in USD were included, meaning funding that the country received from external benefactors such as public or private aid agencies. All monetary flows are valued as total health expenditure per capita (I_3_). Figure 1 depicts inputs and outputs for the first DEA model for humanitarian logistics in Africa.

As outputs, the estimated deaths related to HIV (O_1_) and the number of people with HIV treated by antiretroviral therapy (O_2_), were used to address the input of the total amount of adults (ages 15+) and children (0–14 years). Including the estimated deaths related to HIV as an output factor within DEA requires its integration as a reciprocal value, because otherwise, a large number of deaths are interpreted as efficient by the DEA calculation: Output value for deaths related to HIV by decision making unit (DMU) DMU_n_ in t_m_ = max. deaths related to HIV for all DMU in a dataset in tm—deaths related to HIV of DMU_n_ in tm. A third output is the life expectancy at birth in years (O_3_), aiming to express the effect of the overall health support beyond existing age groups. In order to take populations’ security of supply into account, the number of people using at least basic drinking water services (O_4_) and of people using at least basic sanitation services (O_5_) were used as further outputs. This setup provides an interesting DEA model efficiency perspective of humanitarian operations in the selected African countries, especially from a long-term development perspective regarding the health care sector. This can be valuated as an important contribution regarding time-series as well as at the actor level since a strategic perspective is incorporated, in many cases lacking in existing operations and optimization approaches in the humanitarian area. To validate the inputs and outputs through the available data, this approach follows Dyson et al. (2001) [56]. First, mixing indices and volume measures, as well as integrating percentages, can lead to distortions of the efficiency values. Second, linked input/output values have to be avoided, e.g., when considering volume measures (x) and resulting total costs (p × x). Third, a cross-correlation of the available factors has to be avoided, which was tested before computing the DEA results. As the highest correlation was *r* = 0.47, there is no significant statistical relationship between input and output factors.

### 3.2. Private Non-Profit and Non-Governmental Organizations

**Figure 2:**
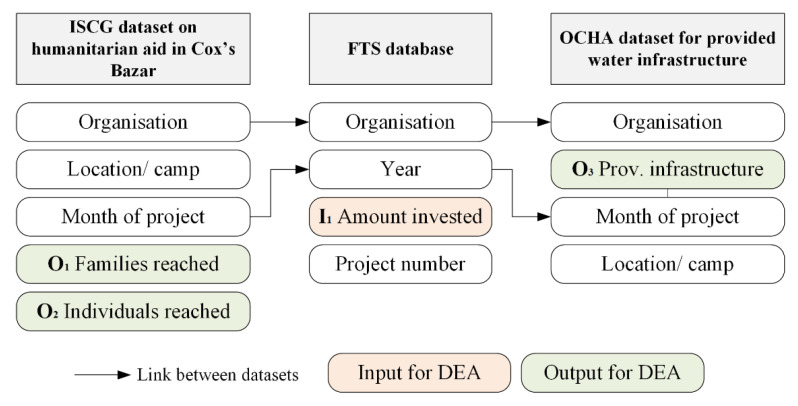
Data sources and data envelopment (DEA) model for the analysis of NPO/non-government organizations (NGO). Note: ISCG (Inter Sector Coordination Group) [60], FTS (Financial Tracking Service), OCHA (United Nations Office for the Coordination of Humanitarian Affairs), I (inputs for DEA model), O (outputs for DEA model).

In order to address the level of private non-profit and non-governmental organizations, a local humanitarian crisis in Bangladesh was examined as a typical refugee camp situation with multiple actors as outlined e.g., by Brankamp [57]. Following an outbreak of violence on 25 August 2017 in Rakhine State, Myanmar, a new massive influx of Rohingya refugees to Cox’s Bazar, Bangladesh started in mid-August 2017. Most of the Rohingya refugees settled in Ukhia and Teknaf Upazilas of Cox’s Bazar, which is a district bordering Myanmar identified as the main entry area for border crossings. The refugee camp is divided into sub-camps with different amounts of families and individuals in need. The following figure summarizes the DEA model for non-profit organizations (NPO) actors applied to Cox’s Bazar refugee camp in Bangladesh and all data sources used to arrive at a meaningful data pool. Datasets related to financial flows were exported from the Financial Tracking Service (FTS), which aims to present a complete picture of all international humanitarian funding flows. It verifies and combines NPO/NGO reports, ensures that data is fully comparable, and publishes it in one database. The invested amount per year was divided into 2 periods in 2017 as the crisis started in August 2017 and 4 quartiles in 2018 (I_1_). Data related to the refugees are provided by the World Bank [58]. The Inter Sector Coordination Group (ISCG) is coordinating the overall Rohingya Refugee Crisis and documents all beneficiaries related to projects of aid organizations, as well as situation reports and maps [59]. The number of beneficiaries is connected to humanitarian organizations, the location, and name of the sub-camp. Further editing was done when linking the beginning and end of a certain project to a time period t_Q3–2017_ until t_Q4–2018_. With this dataset, it was possible to extract two outputs (see Figure 2): O_1_ total beneficiaries reached (families) and O_2_ total beneficiaries reached (individuals). O_3_ represents the amount of water infrastructure provided by NGOs, which was extracted from a dataset that documents the process of providing water infrastructure to all camps. Again, this DEA model setup is typical for a regional crisis situation, and therefore, it represents the second humanitarian area addressing the short-term help for people in need following wars, disasters or other incidents. Therefore, improvement insights regarding overall efficiency are of high interest also in this type of humanitarian engagement in order to increase the impact of humanitarian operations in total.

## 4. Empirical Efficiency Results

### 4.1. Results for National Governments and Agencies

The calculation results have been obtained for the presented input and output data in the timeframe of 2002 to 2015 while applying an output-oriented DEA model with the software BANXIA Frontier Analyst (Banxia Software, Highgate, UK) for the analyzed 34 African countries (for a dataset overview used for case 1, see Table S1). In order to identify whether constant returns to scale (CRS) or variable returns to scale (VRS) has to be applied in the given setup, 14 application runs were conducted for a CCR DEA model calculation (see Table S2) and for the BCC DEA model calculation (see Table S3). The following Table 3 outlines the results by using descriptive statistics. 

In this analysis case on a national level, a series of countries are featuring efficient settings (e.g., Burundi, Eritrea, or Nigeria), whereas low-efficiency levels were attached to e.g., Angola, Mozambique, or Uganda. As the results of the CCR and the BCC model are different, VRS is used for further calculations in this research paper, including bootstrap calculation and a DEA Malmquist index model, see for an example [61]. The differences originate from the countries’ individual allocation of resources, aiming to achieve optimal results. The concept of the most productive scale size (MPSS) is common in the DEA literature, and as the individual performance capability of the countries is different, e.g., different gross domestic product (GDP) and human development index (HDI) ranking, VRS has to be applied. In order to find out about any possibly occurring bias within the dataset, 14 bootstrap calculation runs are conducted with the dataset used above and an output-oriented BCC model. In general, bootstrapping is a statistical methodology where a resampling technique is used to estimate statistics on a population, e.g., the mean or standard deviation. This is achieved by sampling a dataset with replacement. When combining bootstrapping with DEA, the aim is to calculate bias-corrected DEA efficiency scores. The results of these bias-corrected efficiency values for technical efficiency scores, as well as the lower and upper bound, were obtained by using the rDEA package of the software R (R Foundation, Vienna, Austria). The bootstrap algorithm of Simar and Wilson (1998) uses B = 1000 bootstrap iterations, whereby alpha = 5% was set as a confidence interval [62]. Then, the final bias-corrected DEA efficiency scores are calculated as an average value from the B operations. The following Table 4 summarizes the results exemplary for 2015 by presenting arithmetic mean values for the efficiency scores, bias-corrected efficiency scores, as well as lower and upper bound of the 5% confidence interval by iteratively resampling the dataset with replacements. The bootstrap calculations can be found in detail for the years 2014 and 2015 in Table S4.

The results show that the bias-corrected efficiency scores are lower than the original ones, and all values of real efficiency scores are within the lower and upper bound within a confidence interval of 5%. Nevertheless, the exemplary named countries as efficiency leaders as well as countries lacking behind in efficiency of (health) operations are identical (Angola, Mozambique, or Uganda). Aspiring to realize a longitudinal efficiency analysis, an output-oriented DEA Malmquist index model is implemented to analyze the time series data of 34 African countries. To provide further methodological insights regarding usage of specific inputs and outputs, this publication tests six DEA Malmquist index model modifications. Therefore, (I) DEA Malmquist for original model, (II) DEA Malmquist excluding all HIV related factors, and (III) DEA Malmquist excluding infrastructure-related outputs O_4_, O_5_ from the original model are used in analyses I–III. Models IV–VI examine the effect of weighting O_3_ with 5%, 10%, and 15% in the original model (Table 5).

The following Table 6 outlines the results with regard to the Malmquist index values, catchup values, as well as the frontier shift values in an exemplary case for Angola. 

It can be recognized as might be hypothesized for countries of the Global South that most of the efficiency changes originate in the field of frontier shift developments and hence lead to technology improvements in the overall production frontier due to new available technologies and process concepts. This could be an interesting result to be validated with other datasets for comparable countries.

### 4.2. Results for Private Non-Profit and Non-Governmental Organizations

The calculation results have been obtained for the presented input and output data in the timeframe of t_Q3–2017_ until t_Q4–2018_ while applying four input-oriented as well as four output-oriented DEA models with the software BANXIA Frontier Analyst for the analyzed 8 NPO (dataset see also Table S6). The following Table 7 aggregates the modifications calculated for the NPO/NGO model.

The following Table 8 summarizes the results per modification with key figures of descriptive statistics (see also Table S6 and Table S7).

In this case, obviously, efficiency levels are very diverse, as can be expected in contrast to the more aggregate national level in case 1 as the size variation with different NPO/NGO actors is very high. Few actors are efficient in some years (IOM—International Organization for Migration), whereas a number of actors feature quite low efficiency levels (e.g., ACF—Action Contre La Faim, UNHCR—United Nations High Commissioner for Refugees or WVI—Worls Vision International). This requires further analysis as to the exact difference between the actors in such close proximity as with a refugee camp area.

The difference between the efficiency scores of input and output-oriented models signals the necessity of choosing one. As the aim of humanitarian aid is to help people and save financial investment, an output-oriented model is pursued. As the CCR and the BCC model show different efficiency scores, VRS is used for further calculations in this research paper, including bootstrap calculation and DEA window analysis. The differences originate from the extent of human aid provided within the camp. As the individual performance capability of the NPO/NGOs is different (e.g., different size and financial strength of the organizations.), VRS has to be applied. The decision for integration or exclusion of O_3_ is taken by bootstrapping VI and VIII. Although a higher number of factors valorizes the DEA model, the application of Simar and Wilson (1998) [62] bootstrap algorithm with B = 1000 bootstrap iterations and an alpha = 5% as confidence interval showed that the efficiency scores of VI are beyond the lower and upper bound. In contrast, model VIII fulfills the alpha = 5% confidence interval and is therefore used for DEA window analysis.

Furthermore, the highly volatile amounts invested by the NPO/NGO cause a relevant up- or downgrading of individual DMU. In t_Q3–2017_, the International Organization for Migration (IOM) is investing 64.56 times more (9,895,337 $ USD) than the smallest DMU World Vision International with about 153,000 USD. Therefore, IOM and United Nations High Commission for Refugees (investing 38,855,611 $ USD per quartile in 2018) are excluded from further calculations. The following Table 9 summarizes the results of bootstrap calculations for modification VIII (for the whole dataset per quartile and organization, see Table S9).

The effect of integrating highly investing NPO/NGO into the DEA models can also be observed when analyzing the results of DEA window analysis. The modifications including all organizations use eight DMU, and the model ignoring highly investing NPO/NGO used six DMU (see Table 10).

The following Table 11 shows the average efficiencies DEA window analysis modifications (for details on cases I–IV, see Table S10).

## 5. Discussion and Simulation

After elaborating a multi-level and time-series DEA approach for global humanitarian operations and logistics in the previous sections, in a first drafting step, Section 5 deals with the identification of relevant impact factors on the level of efficiency for humanitarian operations. Therefore, the bias-corrected efficiency scores from 2002 to 2015 from the first model related to the national governments and agencies that are used. Malawi was chosen exemplarily for further regression analyses due to its total population of 17 million people (median of 34 African countries: total population of 15 million). The level of efficiency for humanitarian operation is used as the dependent variable, which is influenced by the following independent variables: internal health expenditures, external health expenditures, gross national income (GNI) per capita, life expectancy at birth, and proportion of the rural population. The following Table 12 summarizes the results.

With a view to the logistical aspect of humanitarian operations, the negative correlation between the proportion of the rural population (independent) and level of efficiency for humanitarian operation (dependent) indicates that the complexity of humanitarian operations increases with higher levels of population heterogeneity. In order to support humanitarian operations in countries of the Global South as well as to foster DEA as a method for simulation, the second part of Section 5 aims to develop an ex ante efficiency simulation for humanitarian operations. The general idea is to merge the efficiency values of the DEA approach (as a dependent variable) and the findings of the linear regression analysis with independent variables as well as to increase the original sample size n_sample_ for a simulation sample n_sim_ by B bootstrap iterations. As one bootstrap calculation for both variables, the dependent and independent, as well as resampling solely the independent variable, destroys the link of the DMU and its characteristics, bootstrap iterations are conducted on the DEA model and its efficiency values. Table 13 describes the results of seven regression analyses for six bootstraps with an alpha = 5% confidence interval.

**Figure 3:**
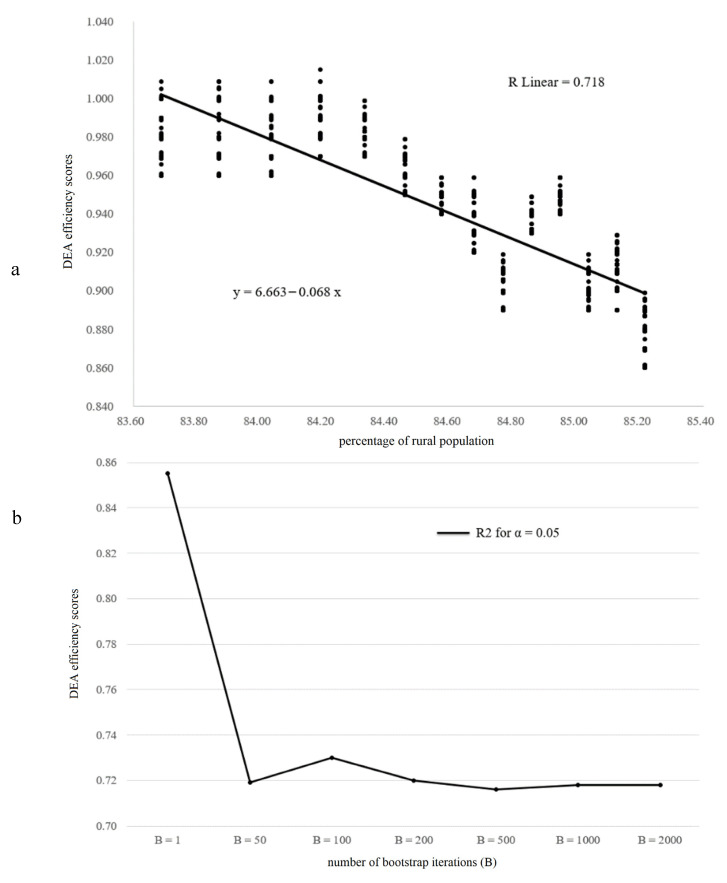
Linear regression model for 2000 bootstraps (upper chart—(**a**)) and development of R^2^ (chart below—(**b**)).

The findings show that the values of the regression analysis stabilize between 500 and 2000 bootstrap iterations. The linear regression equation y = 6.663 − 0.068x of α = 0.05, which can explain 71.8% of the regression model’s variance, is used for further calculations. To illustrate the effect of bootstrapping calculation on the linear regression model, Figure 3 illustrates the linear regression model for 2000 bootstraps and the development of R_2_ for increasing iterations with α = 0.05.

**Figure 4:**
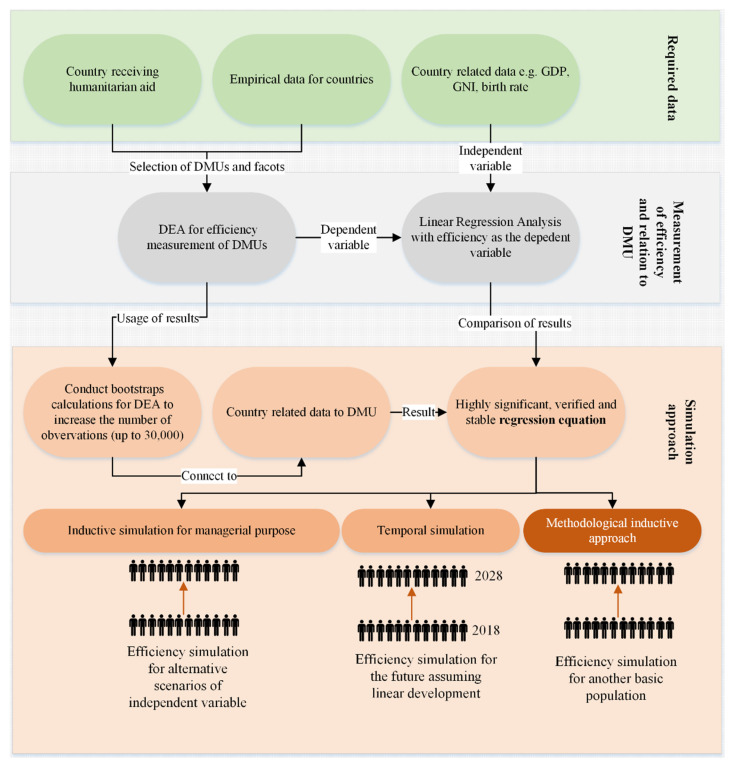
Requirements, techniques, and outcomes for a simulation approach.—Note: GDP (gross domestic product); DMU (Decision making unit); DEA (Data Envelopment Analysis.

With a stable and highly significant linear regression equation, it is now possible to accelerate (1) a managerial approach by focusing on a temporal and an inductive simulation when answering current issues of humanitarian logistics e.g., “What will be the efficiency level for humanitarian aid provided to Malawi, if the proportion of rural population changes?” and (2) a methodological approach answering questions such as e.g., “How will the efficiency curve develop during humanitarian operations in another country?”. Figure 4 illustrates the requirements, techniques, and outcomes for the simulation approach.

**Figure 5:**
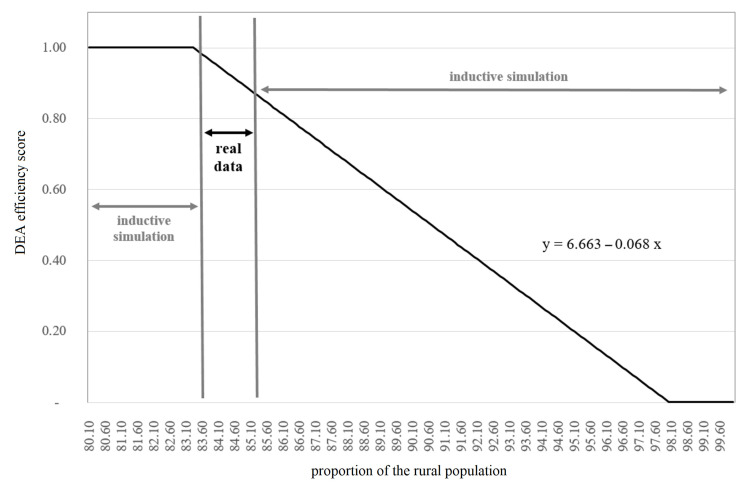
Empirical and simulation data range for a managerial inter-temporal approach.

For a managerial approach, the verified linear regression equation efficiency = 6.663 − 0.068 × proportion of the rural population is applied to determine the total efficiency of humanitarian aid for alternative proportions of the rural population. Thereby, a data area based on real data and beyond this, an inductive simulation data area for scenarios with no real data can be provided. The curve progression indicates that humanitarian operations become significantly inefficient (less than 60% efficiency) when the inputs and outputs of the DEA model remain constant, but the proportion of the rural population increases up to 90%. The same systematic can be applied to temporal simulation when assuming a linear development of the independent variable in the future as well as to other variables tested through regression analysis e.g., internal health expenditures, external health expenditures, GNI per capita, or life expectancy at birth. The following Figure 5 illustrates the real and simulation data for managerial and temporal approach. From this outline, interesting prognosis capabilities might be derived in order to improve the overall efficiency of humanitarian operations.

Within this section, we showed the contribution and value of an in-depth analysis derived from the DEA analysis presented in Section 4. Results from the bootstrap calculation as well as regression analysis show further insights such as for example the role of population heterogeneity—i.e., spatial distribution—for the efficiency of humanitarian logistics operations.

## 6. Conclusions

This paper has shown a way forward in strategic efficiency analysis for humanitarian operations as also addressed for example by Dubey et al. [63] as a relevant field or research in order to improve the humanitarian situation. Specifically, the application of the DEA technique is supported by state-of-the-art time-series model applications on multiple levels as relevant for humanitarian operations. The contribution of this paper consists of the fact that a specified DEA model for existing international data on a national, as well as a regional level with private actors, is developed. Furthermore, the Malmquist index plus window analysis are used in order to cope with the long-time output effects of humanitarian operations. Of special value for further management improvements is the outlined option to analyze and prognose relevant context factors for gaining efficiency in humanitarian operations. For example, the factor of spatial population density and heterogeneity was identified as having an important impact for humanitarian logistics operations.

Limitations of this research include the real-life data restrictions due to limited access for different countries or time series. Therefore, all results address a specific location and time combination and are primarily not transferable. In addition, the applied time-series methods Malmquist index and window analysis also bring about specific restrictions in the analysis such as for example the question of different timeframe perspectives. Further research avenues can be directed at elaborating the dataset and data provision situation as well as the extension of countries analyzed. Furthermore, the suggested regression analysis and prognosis approach can be strengthened by adding more cases and experience to the new concept.

Altogether, efficiency research for humanitarian operations is a demanding but also rewarding field as there is potential to improve our help in need for humans affected by global disaster and crisis situations as outlined by Pettit and Beresford [64] or Beamon and Balcik [55].

## Figures and Tables

**Table 1:** Multiple actor levels in humanitarian operations.

Level of Analysis	Government	Non-Profit/Non-Governmental Organizations (NGO)
**International/ Supra-national**	United Nations (UN) Actors	*Global Actors (Red Cross, Medecines Sans Frontieres—MSF), etc.)*
**National**	*National Governments and Agencies*	Local/Regional/National Actors

Note: Levels written in italic are analyzed in this paper.

**Table 2:** Literature review operations research (OR) methods and efficiency analysis humanitarian operations.

Methodology	Health Care and Civil Security	Humanitarian Operations
OR Methods	Savaser et al. (2018), Transportation optimization for organ transplants in Turkey [13] Decerle et al. (2019), Memetic ant colony optimization algorithm for home health care [27] Grenouilleau et al. (2019), Set partitioning heuristic for home health care routing and scheduling [28] Ng et al. (2018), Meta-heuristics approaches for airside operation research [29] Netjasov, Crnogorac and Pavlović (2019), Network-based simulation model for air traffic management safety performance [30] Janssen, Sharpanskykh and Curran (2019), Agent-based modelling for security and efficiency in airport terminals [31] Ni et al. (2019), Deep belief network and principal component analysis for civil aviation safety evaluation [32]	Noyan and Kahvecioglu (2018), SCM branch-and-cut algorithm for last mile distribution in disaster situations [14] Acimovic and Goentzel J (2016), Models and metrics to assess humanitarian response capacity [33] Roh, Shin and Seo (2018) Warehouse location humanitarian relief logistics [34] Celik and Gumus (2017), Assessment for NGO in humanitarian relief logistics [35] Duque and Sörensen (2011), GRASP metaheuristic for disaster accessibility [36] Wang and Zhang (2019), Agent-based evaluation of humanitarian relief goods supply capability [37] Gralla and Goentzel (2018), Humanitarian transportation planning heuristics [38] Zhang et al. (2018), Procurement and reserves policies humanitarian logistics [39] Cotes and Cantillo (2019), Facility location models for humanitarian relief logistics [40] Carlanda, Goentzel and Montibeller (2018), Private sector agent models in multi-echolon humanitarian supply chains [41]
Efficiency Analysis/DEA	Li, Zhu and Zhuang (2018), DEA efficiency analysis fire protection service in the US [12] Üstün (2016), DEA for disaster resilience capacity [42] Wei et al. (2004) DEA vulnerability assessment in China [43] Nahangi, Chen and McCabe (2019), DEA safety and efficiency evaluation of construction sites [44] Cavalieri, Guccio and Rizzo (2019), DEA for institutional characteristics for healthcare infrastructures [45] Ganji, Rassafi and Bandari (2019), DEA for road safety [46] Egilmez and McAvoy D (2013), DEA Malmquist index for road safety of U.S. states [47] Cook et al. (2019), DEA for performance evaluation in pay-for-performance incentive plans [48] Khushalani and Ozcan (2017), Network DEA for hospital quality care [49] Camposad et al. (2016), DEA for efficiency of health systems in Spain [50] Barakac and Dahooei (2018), DEA-Fuzzy MADM for airlines safety evaluation [51]	Jola-Sanchez et al. (2016), Effect of armed conflicts on humanitarian operations: Total factor productivity and efficiency of rural hospitals [52] DEA: -

Note: SCM (Supply Chain Management), GRAPS (Greedy Randomized Adaptive Search Procedure), DEA (Data Envelopment Analysis), NGO (Non-Governmental Organization), MADM (Multi Attribute Decision Making); grey field indicating current research gap.

**Table 3:** Results for DEA model for national governments and agencies of 2002–2015.

Country	CCR				BCC			
	Min	Mean	Median	SD	Min	Mean	Median	SD
Angola	0.54	0.57	0.56	0.03	0.93	0.95	0.95	0.01
Benin	0.92	0.97	1.00	0.03	0.99	1.00	1.00	0.00
Burkina Faso	0.88	0.97	1.00	0.04	0.96	0.99	1.00	0.02
Burundi	1.00	1.00	1.00	0.00	1.00	1.00	1.00	0.00
C.A.R.	0.81	0.97	1.00	0.06	0.93	0.99	100	0.02
Cameroon	0.85	0.88	0.88	0.02	0.85	0.89	0.89	0.02
Chad	0.62	0.79	0.82	0.10	0.96	0.98	0.98	0.01
Comoros	1.00	1.00	1.00	0.00	1.00	1.00	1.00	0.00
Djibouti	1.00	1.00	1.00	0.00	1.00	1.00	1.00	0.00
Equatorial G.	1.00	1.00	1.00	0.00	1.00	1.00	1.00	0.00
Eritrea	1.00	1.00	1.00	0.00	1.00	1.00	1.00	0.00
Ethiopia	1.00	1.00	1.00	0.00	1.00	1.00	1.00	0.00
Ghana	0.99	1.00	1.00	0.00	0.99	1.00	1.00	0.00
Guinea	0.85	0.98	1.00	0.05	0.97	1.00	1.00	0.01
Guinea-B.	1.00	1.00	1.00	0.00	1.00	1.00	1.00	0.00
Kenya	0.88	0.97	1.00	0.04	0.93	0.99	1.00	0.02
Lesotho	0.93	0.98	0.99	0.02	0.94	0.98	0.99	0.02
Liberia	0.84	0.96	0.99	0.06	0.98	0.99	0.99	0.01
Madagascar	1.00	1.00	1.00	0.00	1.00	1.00	1.00	0.00
Malawi	0.89	0.95	0.94	0.04	0.89	0.95	0.95	0.04
Mali	0.94	0.98	0.99	0.02	0.97	0.99	1.00	0.01
Mauritania	1.00	1.00	1.00	0.00	1.00	1.00	1.00	0.00
Morocco	1.00	1.00	1.00	0.00	1.00	1.00	1.00	0.00
Mozambique	0.47	0.65	0.66	0.08	0.81	0.85	0.85	0.02
Namibia	0.92	0.99	1.00	0.03	1.00	1.00	1.00	0.00
Niger	0.96	1.00	1.00	0.01	1.00	1.00	1.00	0.00
Nigeria	1.00	1.00	1.00	0.00	1.00	1.00	1.00	0.00
Rwanda	0.88	0.98	1.00	0.04	0.92	0.99	1.00	0.03
Senegal	0.96	0.99	1.00	0.02	1.00	1.00	1.00	0.00
Sierra Leone	0.69	0.75	0.76	0.04	0.98	0.99	0.99	0.00
Sudan	0.74	0.87	0.84	0.09	0.99	1.00	1.00	0.00
Togo	0.75	0.93	0.95	0.08	0.97	0.98	0.98	0.01
Uganda	0.62	0.83	0.85	0.13	0.84	0.92	0.89	0.06
Zambia	0.76	0.92	0.97	0.10	0.76	0.93	1.00	0.10

Note: C.A.R (Central African Republic); Equatorial G. (Equatorial Guinea); Guinea-B. (Guinea-Bissau); SD (standard deviation); CCR (DEA model with constant returns to scale); BCC (DEA model with variable returns to scale).

**Table 4:** Results for DEA 14 bootstrap calculations with B = 1000 iterations.

Country	Mean Efficiency Scores	Mean Bias-Corrected Efficiency Scores	Mean Lower Bound	Mean Upper Bound
Angola	0.95	0.98	0.94	1.00
Benin	1.00	0.98	0.97	1.00
Burkina Faso	0.99	0.97	0.96	1.00
Burundi	1.00	0.97	0.95	1.00
C.A.R.	0.99	0.97	0.94	1.00
Cameroon	0.89	0.88	0.87	0.90
Chad	0.98	0.97	0.96	0.98
Comoros	1.00	0.97	0.95	1.00
Djibouti	1.00	0.97	0.95	1.00
Equatorial G.	1.00	0.97	0.95	1.00
Eritrea	1.00	0.97	0.94	1.00
Ethiopia	1.00	0.97	0.95	1.00
Ghana	1.00	0.98	0.96	1.00
Guinea	1.00	0.97	0.96	1.00
Guinea-B.	1.00	0.97	0.95	1.00
Kenya	0.99	0.97	0.95	0.99
Lesotho	0.98	0.97	0.95	0.99
Liberia	0.99	0.97	0.95	1.00
Madagascar	1.00	0.97	0.95	1.00
Malawi	0.95	0.94	0.92	0.96
Mali	0.99	0.98	0.97	1.00
Mauritania	1.00	0.97	0.95	1.00
Morocco	1.00	0.97	0.95	1.00
Mozambique	0.85	0.85	0.84	0.86
Namibia	1.00	0.97	0.95	1.00
Niger	1.00	0.97	0.95	1.00
Nigeria	1.00	0.97	0.95	1.00
Rwanda	0.99	0.97	0.95	1.00
Senegal	1.00	0.98	0.96	1.00
Sierra Leone	0.99	0.98	0.98	0.99
Sudan	1.00	0.98	0.96	1.00
Togo	0.98	0.97	0.96	1.00
Uganda	0.92	0.91	0.89	0.95
Zambia	0.93	0.91	0.90	0.93

Note: C.A.R (Central African Republic); Equatorial G. (Equatorial Guinea); Guinea-B. (Guinea-Bissau).

**Table 5:** Modifications for DEA Malmquist model for national governments and agencies.

DEA Model	Inputs and Outputs Used	Integrated Weights of Factors
I	I_1_, I_2_, I_3_, O_1_, O_2_, O_3_, O_4_, O_5_	none
II	I_2_, I_3_, O_3_, O_4_, O_5_	none
III	I_1_, I_2_, I_3_, O_1_, O_2_, O_3_	none
IV	I_1_, I_2_, I_3_, O_1_, O_2_, O_3_, O_4_, O_5_	5% on O_3_
V	I_1_, I_2_, I_3_, O_1_, O_2_, O_3_, O_4_, O_5_	10% on O_3_
VI	I_1_, I_2_, I_3_, O_1_, O_2_, O_3_, O_4_, O_5_	15% on O_3_

Note: I (input); O (output); Details on inputs and outputs are explained in the text.

**Table 6:** Results of DEA Malmquist modifications for Angola.

Angola	Malmquist Index	Catchup	Frontier Shift	Malmquist Index	Catchup	Frontier Shift	Malmquist Index	Catchup	Frontier Shift
**Modification**	**I**			**II**			**III**		
2002									
2003	1.06	1.00	1.06	1.00	1.01	0.99	1.06	1.00	1.06
2004	1.05	1.00	1.06	0.99	0.99	1.00	1.05	1.00	1.06
2005	0.99	0.99	1.00	1.01	1.01	1.00	0.99	0.99	1.00
2006	1.05	1.00	1.06	0.99	0.99	1.00	1.05	1.00	1.06
2007	1.00	1.00	1.00	1.01	1.01	0.99	1.00	1.00	1.00
2008	1.00	1.00	1.00	1.02	1.01	1.00	1.00	1.00	1.00
2009	0.94	0.99	0.95	1.00	1.00	1.00	0.94	0.99	0.95
2010	1.00	1.00	1.00	1.04	1.03	1.01	1.00	1.00	1.00
2011	0.99	0.99	1.00	0.99	0.99	1.00	0.99	0.99	1.00
2012	1.00	1.00	1.00	1.00	1.00	1.00	1.00	1.00	1.00
2013	0.94	0.99	0.94	1.01	1.00	1.01	0.94	0.99	0.94
2014	0.89	1.00	0.90	1.01	1.01	1.00	0.89	1.00	0.90
2015	1.00	1.00	1.00	1.02	1.01	1.01	1.00	1.00	1.00
**Modification**	**IV**			**V**			**VI**		
2002									
2003	1.06	1.00	1.06	1.05	1.00	1.05	1.05	1.00	1.05
2004	1.05	1.00	1.06	1.05	1.00	1.05	1.04	1.00	1.05
2005	1.00	1.00	1.00	1.00	1.00	1.00	1.00	1.00	1.00
2006	1.05	1.00	1.05	1.05	1.00	1.05	1.04	1.00	1.05
2007	1.00	1.00	1.00	1.00	1.00	1.00	1.00	1.00	1.00
2008	1.00	1.00	1.00	1.00	1.00	1.00	1.00	1.00	1.00
2009	0.95	1.00	0.95	0.95	1.00	0.95	0.95	1.00	0.96
2010	1.00	1.00	1.00	1.00	1.00	1.00	1.01	1.00	1.00
2011	0.99	0.99	1.00	0.99	0.99	1.00	0.99	0.99	1.00
2012	1.00	1.00	1.00	1.00	1.00	1.00	1.00	1.00	1.00
2013	0.94	0.99	0.95	0.94	0.99	0.95	0.94	0.99	0.95
2014	0.90	1.00	0.90	0.90	1.00	0.91	0.91	1.00	0.91
2015	1.00	1.00	1.00	1.00	1.00	1.00	1.01	1.00	1.00

**Table 7:** Modifications of the DEA model for NPO/NGO.

DEA Model	Variables	Input Oriented	Output Oriented
CCR	I_1_, O_1_, O_2_, O_3_	I	V
BCC	I_1_, O_1_, O_2_, O_3_	II	VI
CCR	I_1_, O_1_, O_2_	III	VII
BCC	I_1_, O_1_, O_2_	IV	VIII

Note: CCR (DEA model with constant returns to scale); BCC (DEA model with variable returns to scale); I (input); O (output); Details on inputs and outputs are explained in the text.

**Table 8:** Results for DEA model for NPO/NGO per modification.

NPO	Min	Mean	SD	Min	Mean	Sd	Min	Mean	SD	Min	Mean	SD
**Modification**	**I**			**II**			**III**			**IV**		
ACF	0.02	0.40	0.35	0.04	0.69	0.44	0.02	0.40	0.35	0.04	0.69	0.44
IOM	0.11	0.81	0.33	1.00	1.00	0.00	0.11	0.81	0.33	1.00	1.00	0.00
Oxfam	0.33	0.72	0.29	0.33	0.76	0.26	0.33	0.72	0.29	0.33	0.76	0.26
Save the Children	0.02	0.30	0.37	0.06	0.42	0.38	0.02	0.30	0.37	0.06	0.42	0.38
SI	0.05	0.58	0.40	0.24	0.64	0.33	0.05	0.58	0.40	0.24	0.64	0.33
UN Women	0.09	0.43	0.31	0.32	0.58	0.25	0.09	0.43	0.31	0.32	0.58	0.25
UNHCR	0.00	0.06	0.08	0.00	0.07	0.09	0.00	0.06	0.08	0.00	0.07	0.09
WVI	0.01	0.25	0.30	0.05	0.38	0.44	0.01	0.25	0.30	0.05	0.38	0.44
**Modification**	**V**			**VI**			**VII**			**VIII**		
ACF	0.02	0.40	0.35	0.02	0.68	0.45	0.02	0.40	0.35	0.02	0.68	0.45
IOM	0.11	0.81	0.33	1.00	0.81	0.00	0.11	0.81	0.33	1.00	1.00	0.00
Oxfam	0.33	0.72	0.29	0.42	0.72	0.26	0.33	0.67	0.27	0.42	0.69	0.24
Save the Children	0.02	0.30	0.37	0.05	0.30	0.41	0.02	0.30	0.37	0.05	0.45	0.41
SI	0.05	0.58	0.40	0.08	0.58	0.38	0.05	0.58	0.40	0.08	0.60	0.38
UN Women	0.09	0.43	0.31	0.10	0.43	0.32	0.09	0.43	0.31	0.10	0.47	0.32
UNHCR	0.00	0.06	0.08	0.01	0.06	0.10	0.00	0.06	0.08	0.01	0.13	0.10
WVI	0.01	0.25	0.30	0.03	0.25	0.42	0.01	0.25	0.30	0.03	0.42	0.42

Note: ACF (Action Contre La Faim); IOM (International Organization for Migration); SI (Solidarités International); UN (United Nations); UNHCR (United Nations High Commissioner for Refugees); WVI (World Vision International); SD (standard deviation); NPO (Non Profit Organizations).

**Table 9:** Result for bootstrap calculations with B = 1000 iterations.

Organisation	Mean Efficiency Scores	Mean Bias-Corrected Efficiency Scores	Mean Lower Bound	Mean Upper Bound
ACF	0.75	0.72	0.71	0.78
Oxfam	0.76	0.70	0.59	0.92
Save the Childr.	0.54	0.50	0.32	0.56
SI	0.68	0.56	0.45	0.79
UN Women	0.47	0.32	0.15	0.74
WVI	0.46	0.36	0.26	0.57

Note: ACF (Action Contre La Faim); SI (Solidarités International); UN (United Nations); WVI (World Vision International).

**Table 10:** Modifications of DEA window analysis model for NPO/NGO.

Applied Window Width in DEA Window Analysis	8 DMU	6 DMU
Window width, w = 2	I	III
Window width, w = 3	II	IV

Note: DMU (Decision Making Unit).

**Table 11:** Average efficiencies for DEA window analysis modifications.

**I**	**Q3–2017**	**Q4–2017**	**Q1–2018**	**Q2–2018**	**Q3–2018**	**Q4–2018**	**Mean**
ACF	0.02	0.02	1.00	1.00	1.00	1.00	0.71
IOM	0.37	1.00	1.00	0.71	0.95	1.00	0.87
Oxfam	0.10	1.00	1.00	0.22	1.00	0.48	0.70
Save the Childr.	0.14	0.86	0.06	0.03	0.47	0.07	0.30
SI	0.10	0.76	0.16	0.66	0.08	1.00	0.44
UN Women	0.43	0.46	0.16	0.15	0.07	0.29	0.24
UNHCR	0.03	0.21	0.01	0.17	0.14	0.01	0.11
WVI	0.23	0.56	0.03	0.24	0.06	0.03	0.20
**II**	**Q3–2017**	**Q4–2017**	**Q1–2018**	**Q2–2018**	**Q3–2018**	**Q4–2018**	**Mean**
ACF	0.02	0.02	1.00	1.00	1.00	1.00	0.76
IOM	0.37	1.00	1.00	0.61	0.64	0.94	0.78
Oxfam	0.07	1.00	1.00	0.11	0.81	0.48	0.62
Save the Childr.	0.10	0.72	0.06	0.02	0.23	0.06	0.19
SI	0.07	0.63	0.16	0.44	0.07	1.00	0.36
UN Women	0.09	0.15	0.16	0.12	0.04	0.29	0.13
UNHCR	0.02	0.18	0.01	0.13	0.05	0.00	0.07
WVI	0.03	0.13	0.03	0.17	0.04	0.02	0.08
**III**	**Q3–2017**	**Q4–2017**	**Q1–2018**	**Q2–2018**	**Q3–2018**	**Q4–2018**	**Mean**
ACF	0.06	0.07	0.59	0.28	0.87	1.00	0.47
Oxfam	0.10	0.65	1.00	0.22	1.00	0.48	0.63
Save the Childr.	0.14	1.00	0.05	0.04	0.59	0.08	0.36
SI	0.11	0.94	0.14	0.61	0.08	1.00	0.46
UN Women	0.43	0.51	0.15	0.14	0.07	0.29	0.25
WVI	0.23	0.72	0.02	0.28	0.08	0.03	0.25
**IV**	**Q3–2017**	**Q4–2017**	**Q1–2018**	**Q2–2018**	**Q3–2018**	**Q4–2018**	**Mean**
ACF	0.06	0.07	1.00	0.40	0.79	1.00	0.58
Oxfam	0.10	0.65	1.00	0.11	0.81	0.48	0.57
Save the Childr.	0.14	1.00	0.08	0.02	0.30	0.08	0.26
SI	0.11	0.94	0.17	0.45	0.07	1.00	0.41
UN Women	0.15	0.26	0.16	0.12	0.04	0.29	0.16
WVI	0.10	0.44	0.04	0.21	0.05	0.03	0.15

Note: ACF (Action Contre La Faim); IOM (International Organization for Migration); SI (Solidarités International); UN (United Nations); WVI (World Vision International).

**Table 12:** Results of regression analyses for Malawi.

Data Variable	Dependent Variable: Level of Efficiency for Humanitarian Operation				
Internal health expenditures	0.003 ^***^				
	(0.001)				
External health expenditures		0.004 ^***^			
		(0.001)			
GNI per capita			0.0004 ^***^		
			(0.0001)		
Life expectancy at birth				0.006 ^***^	
				(0.001)	
Proportion of rural population					−0.006 ^***^
					(0.013)
Constant	0.843 ^***^	0.850 ^***^	0.800 ^***^	0.612 ^***^	0.617 ^***^
	(0.018)	(0.016)	(0.023)	(0.055)	(1.063)
Observations	14	14	14	14	14
R^2^	0.668	0.686	0.713	0.731	0.670
Adjusted R^2^	0.640	0.660	0.689	0.709	0.642
Residual Std. Error (df = 12)	0.022	0.021	0.020	0.020	0.022
F Statistic (df = 1; 12)	24.103 ^***^	26.245 ^***^	29.845 ^***^	32.676 ^***^	24.354 ^***^

Note: *p < 0.10; **p < 0.05; ***p < 0.01; regression coefficients (numbers not in parentheses); standard error (numbers in parentheses); GNI (gross national income).

**Table 13:** Results for regression analyses of bootstrap calculations.

Data Variable	Dependent Variable: Efficiency (eff.)—Bootstrap (B) α = 0.05						
	eff.	eff. B = 50	eff. B = 100	eff. B = 200	eff. B = 500	eff. B = 1000	eff. B = 2000
percentage of rural	−0.079 ***	−0.068 ***	−0.069 ***	−0.068 ***	−0.067 ***	−0.068 ***	−0.068 ***
population	(0.009)	(0.002)	(0.001)	(0.001)	(0.001)	(0.0004)	(0.0003)
Constant	7.621 ***	6.674 ***	6.754 ***	6.663 ***	6.645 ***	6.662 ***	6.663 ***
	(0.791)	(0.136)	(0.095)	(0.068)	(0.043)	(0.030)	(0.021)
Observations	14	700	1400	2800	7000	14,000	28,000
R^2^	0.855	0.719	0.730	0.720	0.716	0.718	0.718
Adjusted R^2^	0.843	0.719	0.729	0.719	0.716	0.718	0.718
Resid. Error df	0.016 df = 12	0.020 df = 698	0.019 1398	0.020 2798	0.020 6998	0.019 13998	0.020 27998
F statistic df	71.025 *** 1; 12	1788.258 *** 1; 698	3771.404 *** 1; 1398	7179.596 *** 1; 2798	17,669.820 *** 1; 6998	35,958.140 *** 1; 13,998	71,458.300 *** 1; 27,998

Note: *p < 0.10; **p < 0.05; ***p < 0.01; regression coefficients (numbers not in parentheses); standard error (numbers in parentheses); df (degrees of freedom).

## Data Availability

No data is provided.

## Supplementary Materials

The following are available online at https://www.mdpi.com/1660-4601/18/5/2219/s1, Table S1: Calculation Data 2002–2015 for 34 African countries; Table S2: Results for output oriented CCR DEA model for national governments and agencies; Table S3: Results for output oriented BCC DEA model for national governments and agencies; Table S4: Exemplary results for DEA bootstrap calculations with 1,000 iterations for 2014 and 2015; Table S5: Calculation results for DEA Malmquist Index 2003–2015 (base year 2002) of modification I-VI; Table S6: Calculation Data Q3–2017-Q4–2018 for 8 NGO; Table S7. Results for input-oriented DEA calculations of NGO model; Table S8: Results output-oriented DEA calculations NGO model; Table S9: Results for DEA bootstrap calculations with 1,000 iterations; Table S10. Results for DEA window analysis, window width (w) =2 and w = 3, integrating O3.
