# Supplementary material for: Long-Term Economic Sustainability of Humanitarian Logistics—A Multi-Level and Time-Series Data Envelopment Analysis
Source: Int J Environ Res Public Health. 2021 Feb 24;18(5):2219. doi: 10.3390/ijerph18052219 (PMC7956518; doi:10.3390/ijerph18052219)
Supplement: Supplementary file 1 [file ijerph-18-02219-s001.pdf]

Table S1. Calculation Data 2002-2015 for 34 African countries.

Country	Year	Adults (ages 15+) and children (0-14 years) living with HIV	Total health expenditure per capita in US$	Population, total	AIDS estimated deaths (reciprocal value in period)	People with HIV treated by antiretroviral therapy	Life expectancy at birth, total (years)	People basic drinking water services	People using basic sanitation services
Angola	2002	110,000	30.17	17,572,649	154,700	-	49.34	6,744,151	4,037,301
…									
Angola	2015	280,000	111.57	27,859,305	153,900	6,964,826	61.24	11,423,950	10,984,763
Benin	2002	55,000	20.79	7,295,394	158,900	-	56.03	4,451,494	763,309
…									
Benin	2015	68,000	42.01	10,575,952	142,000	5,287,976	60.64	7,087,716	1,473,242
Burkina Faso	2002	150,000	10.65	12,293,100	157,800	-	51.38	5,826,262	1,327,451
…									
Burkina Faso	2015	94,000	43.33	18,110,624	169,900	10,141,949	59.93	9,753,947	4,080,701
Burundi	2002	110,000	7.87	6,741,569	169,330	67,416	51.98	3,565,472	2,880,769
…									
Burundi	2015	77,000	34.13	10,199,270	169,220	5,609,599	57.09	5,704,872	5,146,584
C.A.R.	2002	180,000	8.70	3,907,612	169,400	-	43.72	2,045,889	647,573
…									
C.A.R.	2015	110,000	23.88	4,546,100	176,000	1,136,525	51.41	2,461,434	1,140,480
Cameroon	2002	420,000	31.32	16,084,886	167,000	-	51.06	9,118,056	6,367,189
…									
Cameroon	2015	520,000	68.65	22,834,522	50,000	7,535,392	57.58	14,906,368	8,867,783
Chad	2002	98,000	18.16	9,001,689	166,000	-	47.64	3,551,210	885,372
…									

Chad	2015	110,000	40.87	14,009,413	178,100	7,565,083	52.58	5,959,269	1,337,673
Comoros	2002	200	50.36	569,479	178,800	-	59.62	489,792	158,692
…									
Comoros	2015	200	64.44	777,424	178,900	171,033	63.47	650,713	265,615
Djibouti	2002	12,000	48.60	746,221	178,400	-	57.23	560,679	380,731
…									
Djibouti	2015	9,200	100.54	927,414	100,000	194,757	62.26	713,390	476,487
Equatorial G.	2002	16,000	31.54	666,407	170,500	-	53.42	326,042	528,427
…									
Equatorial G.	2015	48,000	284.07	1,175,389	182,000	258,586	57.43	582,876	876,111
Eritrea	2002	19,000	14.01	3,614,639	176,000	-	56.48	615,349	294,049
…									
Eritrea	2015	14,000	38.56	4,913,874	185,000	2,948,324	64.64	947,903	553,181
Ethiopia	2002	1,000,000	5.91	70,497,192	188,900	-	53.35	13,857,278	2,565,448
…									
Ethiopia	2015	620,000	27.99	99,873,033	182,900	61,921,280	65.04	39,074,587	7,069,930
Ghana	2002	300,000	28.39	19,924,522	139,000	-	57.41	13,198,873	2,310,557
…									
Ghana	2015	310,000	99.94	27,582,821	124,000	7,171,533	62.45	21,459,242	3,938,691
Guinea	2002	90,000	25.98	9,137,345	167,000	-	51.45	5,133,787	985,789
…									
Guinea	2015	110,000	31.39	12,091,533	187,200	3,748,375	59.42	8,146,374	2,654,366
Guinea-B.	2002	23,000	18.24	1,293,523	-	-	52.64	711,298	170,192
…									

Guinea-B.	2015	39,000	51.94	1,770,526	164,000	424,926	57.00	1,225,144	380,061
Kenya	2002	1,500,000	22.79	33,214,009	184,000	-	52.57	15,972,626	10,237,787
…									
Kenya	2015	1,500,000	83.15	47,236,259	143,000	28,814,118	66.70	27,612,794	14,097,510
Lesotho	2002	220,000	23.84	1,902,312	184,600	-	46.39	1,275,936	232,067
…									
Lesotho	2015	300,000	114.77	2,174,645	151,000	1,109,069	53.75	1,556,891	952,301
Liberia	2002	62,000	6.47	3,062,863	170,300	-	52.81	1,924,809	416,649
…									
Liberia	2015	41,000	118.43	4,499,621	174,600	809,932	62.01	3,145,257	759,962
Madagascar	2002	16,000	18.52	16,765,117	178,100	-	59.72	6,416,782	872,564
…									
Madagascar	2015	28,000	26.66	24,234,088	173,000	969,364	65.54	12,267,632	2,349,172
Malawi	2002	840,000	16.96	12,013,711	178,300	-	47.17	6,450,920	4,289,248
…									
Malawi	2015	1,000,000	52.53	17,573,607	179,900	10,368,428	62.66	11,808,600	7,649,722
Mali	2002	96,000	15.27	11,638,929	179,220	-	49.54	6,124,000	2,435,453
…									
Mali	2015	130,000	57.68	17,467,905	179,150	4,891,013	57.48	12,973,072	5,461,923
Mauritania	2002	13,000	25.09	2,873,228	179,330	-	60.25	1,608,924	748,828
…									
Mauritania	2015	10,000	58.29	4,182,341	177,400	1,003,762	63.08	2,911,230	1,866,187
Morocco	2002	12,000	63.55	29,512,368	175,300	-	69.74	19,691,408	20,932,342
…									

Morocco	2015	20,000	161.36	34,803,322	132,000	16,009,528	75.57	28,876,315	29,048,964
Mozambique	2002	1,100,000	10.12	19,139,658	175,000	-	49.41	4,820,240	2,593,718
…									
Mozambique	2015	2,000,000	52.37	28,010,691	177,100	10,924,169	57.71	13,237,501	6,599,524
Namibia	2002	160,000	182.66	1,962,147	178,100	-	54.15	1,518,246	567,839
…									
Namibia	2015	190,000	462.27	2,425,561	178,200	1,843,426	63.78	1,911,032	820,731
Niger	2002	40,000	11.34	12,206,002	179,360	-	51.12	4,756,681	833,759
…									
Niger	2015	36,000	32.35	19,896,965	91,000	6,963,938	59.67	9,119,849	2,574,136
Nigeria	2002	2,700,000	16.35	128,666,710	177,400	-	46.83	63,193,101	46,101,858
…									
Nigeria	2015	3,000,000	106.98	181,181,744	166,800	48,919,071	52.99	122,016,787	59,066,982
Rwanda	2002	220,000	11.75	8,536,205	161,700	-	50.97	4,124,895	4,014,149
…									
Rwanda	2015	220,000	81.67	11,629,553	167,000	8,489,574	66.70	6,595,132	7,250,773
Senegal	2002	42,000	26.96	10,396,861	168,600	103,969	58.67	6,596,060	4,136,314
…									
Senegal	2015	45,000	40.30	14,976,994	164,300	6,140,568	66.78	11,260,997	7,242,595
Sierra Leone	2002	32,000	27.72	4,957,216	133,000	-	40.66	2,003,619	527,710
…									
Sierra Leone	2015	61,000	162.83	7,237,025	128,000	1,664,516	51.42	4,204,089	1,051,966
Sudan	2002	21,000	20.24	28,679,565	133,000	-	59.18	12,463,077	6,016,012
…									

Sudan	2015	47,000	154.73	38,647,803	148,300	3,478,302	64.26	22,775,710	13,366,770
Togo	2002	110,000	11.67	5,251,472	-	-	53.71	2,505,892	595,162
…									
Togo	2015	110,000	42.21	7,416,802	121,000	2,818,385	59.95	4,659,060	1,034,531
Uganda	2002	830,000	26.46	25,718,048	146,300	514,361	49.24	7,988,056	4,089,567
…									
Uganda	2015	1,300,000	64.42	40,144,870	131,000	22,079,679	59.58	15,624,972	7,688,126
Zambia	2002	890,000	35.07	11,120,409	143,300	-	46.33	5,585,546	2,961,659
…									
Zambia	2015	1,100,000	86.21	16,100,587	133,000	11,270,411	61.40	9,857,634	5,009,184

Table S2. Results for output oriented CCR DEA model for national governments and agencies.

Country	2002	2003	2004	2005	2006	2007	2008	2009	2010	2011	2012	2013	2014	2015
Angola	0.63	0.62	0.60	0.59	0.56	0.56	0.54	0.55	0.58	0.55	0.54	0.54	0.56	0.58
Benin	0.93	0.92	0.92	0.96	0.95	0.96	1.00	1.00	1.00	1.00	1.00	1.00	1.00	1.00
Burkina Faso	0.93	0.90	0.88	1.00	1.00	0.92	1.00	1.00	1.00	0.99	1.00	1.00	1.00	1.00
Burundi	1.00	1.00	1.00	1.00	1.00	1.00	1.00	1.00	1.00	1.00	1.00	1.00	1.00	1.00
C.A.R.	0.83	0.81	0.99	0.99	1.00	1.00	1.00	1.00	1.00	1.00	1.00	1.00	1.00	1.00
Cameroon	0.86	0.85	0.88	0.91	0.91	0.91	0.90	0.90	0.89	0.91	0.88	0.87	0.85	0.85
Chad	0.62	0.81	0.64	0.64	0.68	0.70	0.87	0.90	0.91	0.82	0.85	0.82	0.84	0.92
Comoros	1.00	1.00	1.00	1.00	1.00	1.00	1.00	1.00	1.00	1.00	1.00	1.00	1.00	1.00
Djibouti	1.00	1.00	1.00	1.00	1.00	1.00	1.00	1.00	1.00	1.00	1.00	1.00	1.00	1.00
Equatorial G.	1.00	1.00	1.00	1.00	1.00	1.00	1.00	1.00	1.00	1.00	1.00	1.00	1.00	1.00
Eritrea	1.00	1.00	1.00	1.00	1.00	1.00	1.00	1.00	1.00	1.00	1.00	1.00	1.00	1.00
Ethiopia	1.00	1.00	1.00	1.00	1.00	1.00	1.00	1.00	1.00	1.00	1.00	1.00	1.00	1.00
Ghana	1.00	1.00	1.00	1.00	1.00	1.00	1.00	1.00	1.00	1.00	1.00	1.00	1.00	0.99
Guinea	0.85	0.86	0.94	1.00	1.00	1.00	1.00	1.00	1.00	1.00	1.00	1.00	1.00	1.00
Guinea-B.	1.00	1.00	1.00	1.00	1.00	1.00	1.00	1.00	1.00	1.00	1.00	1.00	1.00	1.00
Kenya	0.88	0.90	0.91	0.96	1.00	1.00	1.00	1.00	1.00	1.00	1.00	1.00	1.00	1.00

Lesotho	1.00	0.98	0.94	0.93	0.96	1.00	1.00	1.00	1.00	1.00	0.99	0.98	0.97	0.97
Liberia	1.00	1.00	1.00	1.00	1.00	1.00	1.00	0.97	0.98	0.97	0.92	0.90	0.84	0.84
Madagascar	1.00	1.00	1.00	1.00	1.00	1.00	1.00	1.00	1.00	1.00	1.00	1.00	1.00	1.00
Malawi	0.89	0.91	0.90	0.94	0.93	0.90	0.94	0.94	0.97	0.98	1.00	1.00	1.00	1.00
Mali	0.94	0.95	0.99	0.97	1.00	1.00	1.00	1.00	1.00	1.00	0.99	0.97	0.95	0.96
Mauritania	1.00	1.00	1.00	1.00	1.00	1.00	1.00	1.00	1.00	1.00	1.00	1.00	1.00	1.00
Morocco	1.00	1.00	1.00	1.00	1.00	1.00	1.00	1.00	1.00	1.00	1.00	1.00	1.00	1.00
Mozambique	0.64	0.47	0.54	0.55	0.59	0.67	0.68	0.66	0.69	0.72	0.66	0.76	0.74	0.76
Namibia	0.92	0.92	1.00	1.00	1.00	1.00	1.00	1.00	1.00	1.00	1.00	1.00	1.00	1.00
Niger	1.00	0.96	1.00	1.00	1.00	1.00	1.00	1.00	1.00	1.00	1.00	1.00	1.00	1.00
Nigeria	1.00	1.00	1.00	1.00	1.00	1.00	1.00	1.00	1.00	1.00	1.00	1.00	1.00	1.00
Rwanda	0.95	0.88	0.93	1.00	1.00	1.00	1.00	1.00	1.00	1.00	1.00	1.00	1.00	1.00
Senegal	1.00	0.96	0.96	1.00	1.00	1.00	1.00	1.00	1.00	1.00	1.00	1.00	1.00	1.00
Sierra Leone	0.72	0.78	0.78	0.76	0.73	0.79	0.78	0.78	0.79	0.77	0.74	0.70	0.69	0.70
Sudan	1.00	1.00	1.00	0.99	0.83	0.77	0.82	0.83	0.85	0.82	0.87	0.83	0.88	0.74
Togo	0.75	0.77	0.86	1.00	1.00	1.00	0.98	1.00	0.96	0.96	0.93	0.89	0.94	0.95
Uganda	1.00	1.00	1.00	1.00	0.88	0.76	0.76	0.70	0.64	0.62	0.71	0.83	0.86	0.88
Zambia	0.76	0.76	0.76	0.84	0.85	1.00	0.98	0.99	0.97	0.98	0.97	0.98	0.99	1.00

Table S3. Results for output oriented BCC DEA model for national governments and agencies.

Country	2002	2003	2004	2005	2006	2007	2008	2009	2010	2011	2012	2013	2014	2015
Angola	0.97	0.97	0.96	0.96	0.96	0.96	0.95	0.95	0.95	0.94	0.94	0.93	0.93	0.93
Benin	1.00	0.99	1.00	1.00	0.99	0.99	1.00	1.00	1.00	1.00	1.00	1.00	1.00	1.00
Burkina Faso	0.96	0.96	0.96	1.00	1.00	0.98	1.00	1.00	1.00	1.00	1.00	1.00	1.00	1.00
Burundi	1.00	1.00	1.00	1.00	1.00	1.00	1.00	1.00	1.00	1.00	1.00	1.00	1.00	1.00
C.A.R.	0.94	0.93	1.00	1.00	1.00	1.00	1.00	1.00	1.00	1.00	1.00	1.00	1.00	1.00
Cameroon	0.88	0.88	0.89	0.91	0.91	0.91	0.90	0.90	0.90	0.91	0.89	0.88	0.85	0.85
Chad	0.97	0.98	0.96	0.96	0.97	0.97	0.98	0.99	1.00	0.99	0.99	0.98	0.98	0.99
Comoros	1.00	1.00	1.00	1.00	1.00	1.00	1.00	1.00	1.00	1.00	1.00	1.00	1.00	1.00
Djibouti	1.00	1.00	1.00	1.00	1.00	1.00	1.00	1.00	1.00	1.00	1.00	1.00	1.00	1.00
Equatorial G.	1.00	1.00	1.00	1.00	1.00	1.00	1.00	1.00	1.00	1.00	1.00	1.00	1.00	1.00
Eritrea	1.00	1.00	1.00	1.00	1.00	1.00	1.00	1.00	1.00	1.00	1.00	1.00	1.00	1.00
Ethiopia	1.00	1.00	1.00	1.00	1.00	1.00	1.00	1.00	1.00	1.00	1.00	1.00	1.00	1.00
Ghana	1.00	1.00	1.00	1.00	1.00	1.00	1.00	1.00	1.00	1.00	1.00	1.00	1.00	0.99
Guinea	0.97	0.98	0.99	1.00	1.00	1.00	1.00	1.00	1.00	1.00	1.00	1.00	1.00	1.00
Guinea-B.	1.00	1.00	1.00	1.00	1.00	1.00	1.00	1.00	1.00	1.00	1.00	1.00	1.00	1.00
Kenya	0.93	0.95	0.97	1.00	1.00	1.00	1.00	1.00	1.00	1.00	1.00	1.00	1.00	1.00
Lesotho	1.00	0.99	0.95	0.94	0.97	1.00	1.00	1.00	1.00	1.00	1.00	0.98	0.97	0.97
Liberia	1.00	1.00	1.00	1.00	1.00	1.00	1.00	0.98	0.98	0.99	0.99	0.99	0.98	0.98
Madagascar	1.00	1.00	1.00	1.00	1.00	1.00	1.00	1.00	1.00	1.00	1.00	1.00	1.00	1.00
Malawi	0.89	0.91	0.90	0.95	0.93	0.91	0.94	0.95	0.97	0.98	1.00	1.00	1.00	1.00
Mali	0.99	0.99	1.00	1.00	1.00	1.00	1.00	1.00	1.00	1.00	1.00	0.99	0.97	0.97
Mauritania	1.00	1.00	1.00	1.00	1.00	1.00	1.00	1.00	1.00	1.00	1.00	1.00	1.00	1.00
Morocco	1.00	1.00	1.00	1.00	1.00	1.00	1.00	1.00	1.00	1.00	1.00	1.00	1.00	1.00
Mozambique	0.91	0.84	0.84	0.81	0.83	0.85	0.86	0.86	0.86	0.86	0.85	0.86	0.85	0.85
Namibia	1.00	1.00	1.00	1.00	1.00	1.00	1.00	1.00	1.00	1.00	1.00	1.00	1.00	1.00
Niger	1.00	1.00	1.00	1.00	1.00	1.00	1.00	1.00	1.00	1.00	1.00	1.00	1.00	1.00
Nigeria	1.00	1.00	1.00	1.00	1.00	1.00	1.00	1.00	1.00	1.00	1.00	1.00	1.00	1.00
Rwanda	0.96	0.92	0.96	1.00	1.00	1.00	1.00	1.00	1.00	1.00	1.00	1.00	1.00	1.00
Senegal	1.00	1.00	1.00	1.00	1.00	1.00	1.00	1.00	1.00	1.00	1.00	1.00	1.00	1.00
Sierra Leone	1.00	1.00	0.99	0.99	0.99	0.99	0.99	0.99	0.99	0.99	0.99	0.98	0.98	0.98
Sudan	1.00	1.00	1.00	1.00	1.00	0.99	1.00	1.00	1.00	0.99	1.00	0.99	1.00	0.99
Togo	0.98	0.98	0.99	1.00	1.00	1.00	0.98	1.00	0.97	0.98	0.98	0.97	0.97	0.98
Uganda	1.00	1.00	1.00	1.00	1.00	0.87	0.87	0.87	0.84	0.86	0.87	0.89	0.89	0.89
Zambia	0.76	0.77	0.77	0.84	0.85	1.00	1.00	1.00	1.00	1.00	1.00	1.00	1.00	1.00

Table S4. Exemplary results for DEA bootstrap calculations with 1,000 iterations for 2014 and 2015.

2014	Efficiency scores	Bias-corrected efficiency scores	Lower bound	Upper bound	2015	Efficiency scores	Bias-corrected efficiency scores	Lower bound	Upper bound
Angola	0.93	0.99	0.92	1.00	Angola	0.93	0.91	0.89	1.00
Benin	1.00	0.98	0.97	1.00	Benin	1.00	0.98	0.96	1.00
Burkina Faso	1.00	0.98	0.97	1.00	Burkina Faso	1.00	0.98	0.96	1.00
Burundi	1.00	0.97	0.94	1.00	Burundi	1.00	0.97	0.94	1.00
C.A.R.	1.00	0.97	0.95	1.00	C.A.R.	1.00	0.97	0.95	1.00
Cameroon	0.85	0.84	0.83	0.86	Cameroon	0.85	0.84	0.83	0.87
Chad	0.98	0.97	0.96	1.00	Chad	0.99	0.98	0.97	0.99
Comoros	1.00	0.97	0.95	0.98	Comoros	1.00	0.97	0.95	1.00
Djibouti	1.00	0.97	0.94	1.00	Djibouti	1.00	0.97	0.95	1.00
Equatorial G.	1.00	0.97	0.94	1.00	Equatorial G.	1.00	0.97	0.95	1.00
Eritrea	1.00	0.97	0.94	1.00	Eritrea	1.00	0.97	0.94	1.00
Ethiopia	1.00	0.97	0.94	1.00	Ethiopia	1.00	0.97	0.94	1.00
Ghana	1.00	0.98	0.96	1.00	Ghana	0.99	0.97	0.96	1.00
Guinea	1.00	0.97	0.95	1.00	Guinea	1.00	0.98	0.95	1.00
Guinea-B.	1.00	0.97	0.94	1.00	Guinea-B.	1.00	0.97	0.94	1.00
Kenya	1.00	0.98	0.96	1.00	Kenya	1.00	0.97	0.95	1.00
Lesotho	0.97	0.95	0.94	1.00	Lesotho	0.97	0.95	0.94	0.98
Liberia	0.98	0.97	0.96	0.98	Liberia	0.98	0.97	0.97	0.99
Madagascar	1.00	0.97	0.95	0.99	Madagascar	1.00	0.97	0.94	1.00
Malawi	1.00	0.98	0.95	1.00	Malawi	1.00	0.98	0.95	1.00
Mali	0.97	0.96	0.95	1.00	Mali	0.97	0.97	0.94	1.00
Mauritania	1.00	0.97	0.95	0.98	Mauritania	1.00	0.98	0.97	1.00
Morocco	1.00	0.97	0.95	1.00	Morocco	1.00	0.97	0.94	1.00
Mozambique	0.85	0.85	0.84	0.86	Mozambique	0.85	0.85	0.84	0.87
Namibia	1.00	0.97	0.95	1.00	Namibia	1.00	0.97	0.94	1.00
Niger	1.00	0.97	0.95	1.00	Niger	1.00	0.97	0.94	1.00
Nigeria	1.00	0.97	0.95	1.00	Nigeria	1.00	0.97	0.95	1.00
Rwanda	1.00	0.97	0.95	1.00	Rwanda	1.00	0.97	0.95	1.00
Senegal	1.00	0.97	0.95	1.00	Senegal	1.00	0.97	0.94	1.00
Sierra Leone	0.98	0.98	0.97	0.99	Sierra Leone	0.98	0.98	0.97	0.98
Sudan	1.00	0.98	0.97	1.00	Sudan	0.99	0.97	0.96	1.00
Togo	0.97	0.96	0.95	0.98	Togo	0.98	0.96	0.95	0.98
Uganda	0.89	0.88	0.87	0.90	Uganda	0.89	0.88	0.87	0.90
Zambia	1.00	0.98	0.96	1.00	Zambia	1.00	0.98	0.96	1.00

Table S5. Calculation results for DEA Malmquist Index 2003-2015 (base year 2002) of modification I-VI.

Year	Country	(I) Malmquist index	(II) Malmquist index	(III) Malmquist index	(IV) Malmquist index	(V) Malmquist index	(VI) Malmquist index
2002	Angola						
2003	Angola	1.06	1.00	1.06	1.06	1.05	1.05
2004	Angola	1.05	0.99	1.05	1.05	1.05	1.04
2005	Angola	0.99	1.01	0.99	1.00	1.00	1.00
2006	Angola	1.05	0.99	1.05	1.05	1.05	1.04
2007	Angola	1.00	1.01	1.00	1.00	1.00	1.00
2008	Angola	1.00	1.02	1.00	1.00	1.00	1.00
2009	Angola	0.94	1.00	0.94	0.95	0.95	0.95
2010	Angola	1.00	1.04	1.00	1.00	1.00	1.01
2011	Angola	0.99	0.99	0.99	0.99	0.99	0.99
2012	Angola	1.00	1.00	1.00	1.00	1.00	1.00
2013	Angola	0.94	1.01	0.94	0.94	0.94	0.94
2014	Angola	0.89	1.01	0.89	0.90	0.90	0.91
2015	Angola	1.00	1.02	1.00	1.00	1.00	1.01
2002	Benin						
2003	Benin	1.03	0.98	1.05	1.03	1.03	1.02
2004	Benin	1.03	0.99	1.05	1.03	1.03	1.02
2005	Benin	1.00	1.01	1.00	1.00	1.00	1.00
2006	Benin	1.03	1.00	1.05	1.03	1.03	1.03
2007	Benin	0.99	0.98	1.00	0.99	0.99	0.99
2008	Benin	1.13	1.01	1.15	1.13	1.13	1.13
2009	Benin	1.12	0.99	1.12	1.11	1.11	1.11
2010	Benin	1.11	1.00	1.11	1.11	1.10	1.10
2011	Benin	1.11	0.98	1.12	1.10	1.10	1.10
2012	Benin	1.01	1.02	1.04	1.01	1.01	1.01
2013	Benin	0.98	0.99	0.94	0.98	0.98	0.99
2014	Benin	0.98	1.00	0.91	0.98	0.99	0.99
2015	Benin	1.05	1.01	1.00	1.05	1.05	1.05
2002	Burkina Faso						
2003	Burkina Faso	1.10	0.98	1.12	1.10	1.10	1.09
2004	Burkina Faso	1.16	0.97	1.20	1.16	1.16	1.15
2005	Burkina Faso	1.30	1.00	1.31	1.30	1.29	1.28
2006	Burkina Faso	1.27	0.99	1.29	1.26	1.25	1.24
2007	Burkina Faso	1.12	0.97	1.13	1.11	1.11	1.10
2008	Burkina Faso	1.17	1.01	1.17	1.17	1.17	1.16
2009	Burkina Faso	1.13	1.01	1.13	1.13	1.13	1.12
2010	Burkina Faso	1.13	1.00	1.13	1.13	1.13	1.13
2011	Burkina Faso	1.10	1.01	1.10	1.09	1.09	1.08
2012	Burkina Faso	1.04	1.00	1.04	1.04	1.05	1.05
2013	Burkina Faso	1.01	1.01	0.99	1.01	1.01	1.01
2014	Burkina Faso	0.99	1.02	0.96	0.99	0.99	1.00

2015	Burkina Faso	1.06	1.02	1.02	1.06	1.06	1.06
2002	Burundi						
2003	Burundi	0.97	0.96	0.97	0.97	0.97	0.98
2004	Burundi	1.58	0.99	1.58	1.56	1.55	1.53
2005	Burundi	1.16	0.98	1.16	1.15	1.14	1.14
2006	Burundi	0.78	0.71	1.05	0.79	0.81	0.83
2007	Burundi	1.21	1.01	1.23	1.20	1.19	1.19
2008	Burundi	0.81	0.59	1.07	0.83	0.84	0.86
2009	Burundi	0.63	0.73	0.98	0.66	0.69	0.72
2010	Burundi	0.89	0.87	1.01	0.90	0.90	0.91
2011	Burundi	1.12	1.08	1.01	1.11	1.11	1.10
2012	Burundi	1.24	0.93	1.01	1.22	1.21	1.19
2013	Burundi	0.93	1.16	0.95	0.93	0.93	0.94
2014	Burundi	1.11	1.11	0.94	1.11	1.10	1.10
2015	Burundi	0.92	0.93	1.00	0.93	0.93	0.94
2002	C.A.R.						
2003	C.A.R.	1.05	0.99	1.05	1.05	1.04	1.04
2004	C.A.R.	1.04	1.01	1.04	1.04	1.04	1.04
2005	C.A.R.	0.97	0.90	0.97	0.97	0.96	0.96
2006	C.A.R.	1.01	1.01	1.02	1.01	1.01	1.01
2007	C.A.R.	0.62	0.57	0.97	0.65	0.68	0.71
2008	C.A.R.	0.95	0.97	1.01	0.95	0.95	0.95
2009	C.A.R.	0.99	0.89	0.95	0.99	0.99	0.99
2010	C.A.R.	1.01	1.04	1.05	1.01	1.02	1.02
2011	C.A.R.	1.00	0.98	0.98	0.99	0.99	0.99
2012	C.A.R.	1.00	1.02	1.01	1.01	1.01	1.01
2013	C.A.R.	0.97	1.02	0.98	0.98	0.98	0.98
2014	C.A.R.	1.01	0.99	0.97	1.01	1.01	1.01
2015	C.A.R.	1.00	1.01	1.01	1.01	1.01	1.01
2002	Cameroon						
2003	Cameroon	1.01	0.99	1.05	1.01	1.01	1.01
2004	Cameroon	1.03	1.00	1.06	1.03	1.03	1.02
2005	Cameroon	1.02	1.01	1.03	1.02	1.02	1.01
2006	Cameroon	1.03	1.01	1.06	1.03	1.02	1.02
2007	Cameroon	1.02	0.98	1.01	1.02	1.02	1.02
2008	Cameroon	0.99	0.97	1.02	0.99	0.99	0.99
2009	Cameroon	1.01	1.03	0.97	1.01	1.01	1.01
2010	Cameroon	1.02	1.01	1.01	1.02	1.02	1.02
2011	Cameroon	1.03	1.03	1.00	1.03	1.03	1.03
2012	Cameroon	1.00	1.00	1.00	1.00	1.00	1.00
2013	Cameroon	0.98	0.99	0.96	0.98	0.98	0.99
2014	Cameroon	0.94	1.00	0.92	0.94	0.95	0.95
2015	Cameroon	1.04	1.03	1.02	1.04	1.04	1.04
2002	Chad						

2003	Chad	1.40	0.99	1.40	1.40	1.40	1.41
2004	Chad	1.04	0.96	1.04	1.04	1.03	1.03
2005	Chad	1.00	0.99	1.00	1.00	1.00	1.00
2006	Chad	1.06	1.00	1.06	1.06	1.05	1.05
2007	Chad	1.01	1.01	1.01	1.01	1.01	1.01
2008	Chad	1.06	1.00	1.06	1.06	1.06	1.07
2009	Chad	1.05	1.01	1.05	1.06	1.06	1.06
2010	Chad	1.06	1.01	1.06	1.06	1.06	1.06
2011	Chad	1.00	1.00	1.00	1.00	1.00	1.00
2012	Chad	1.00	1.00	1.00	1.00	1.00	1.00
2013	Chad	0.95	1.02	0.95	0.95	0.95	0.95
2014	Chad	0.90	1.00	0.89	0.91	0.91	0.92
2015	Chad	1.01	1.02	1.01	1.01	1.02	1.02
2002	Comoros						
2003	Comoros	1.03	1.00	1.03	1.03	1.03	1.03
2004	Comoros	1.03	1.00	1.03	1.03	1.03	1.02
2005	Comoros	1.03	1.00	1.00	1.10	1.10	1.10
2006	Comoros	1.24	1.00	1.24	1.23	1.22	1.21
2007	Comoros	1.31	1.00	1.31	1.29	1.28	1.27
2008	Comoros	1.11	1.00	1.11	1.10	1.10	1.09
2009	Comoros	1.00	1.00	1.00	1.00	1.00	1.00
2010	Comoros	1.17	1.00	1.17	1.16	1.15	1.15
2011	Comoros	1.13	1.00	1.13	1.13	1.12	1.11
2012	Comoros	1.00	1.00	1.00	1.00	1.00	1.00
2013	Comoros	1.09	1.00	1.09	1.08	1.08	1.07
2014	Comoros	1.00	1.01	1.00	1.00	1.00	1.00
2015	Comoros	1.00	1.00	1.00	1.00	1.00	1.00
2002	Djibouti						
2003	Djibouti	1.41	1.00	1.40	1.40	1.40	1.40
2004	Djibouti	1.00	1.00	1.01	1.00	1.00	1.00
2005	Djibouti	1.34	1.00	1.34	1.33	1.31	1.30
2006	Djibouti	1.22	1.00	1.22	1.21	1.20	1.19
2007	Djibouti	1.12	1.00	1.12	1.12	1.11	1.11
2008	Djibouti	1.09	1.00	1.09	1.08	1.08	1.07
2009	Djibouti	1.00	1.00	1.00	1.00	1.00	1.00
2010	Djibouti	1.02	1.00	1.02	1.02	1.02	1.01
2011	Djibouti	1.01	1.00	1.00	1.01	1.01	1.01
2012	Djibouti	0.94	1.06	1.00	0.94	0.95	0.95
2013	Djibouti	1.04	0.93	0.97	1.04	1.03	1.03
2014	Djibouti	0.89	0.88	0.93	0.90	0.90	0.91
2015	Djibouti	0.97	0.95	1.00	0.98	0.98	0.98
2002	Equatorial G.						
2003	Equatorial G.	1.03	1.00	1.03	1.03	1.03	1.03
2004	Equatorial G.	1.20	1.00	1.09	1.19	1.18	1.17

2005	Equatorial G.	1.06	0.89	1.15	1.06	1.06	1.06
2006	Equatorial G.	0.87	0.82	1.06	0.88	0.89	0.90
2007	Equatorial G.	1.03	0.94	1.00	1.03	1.03	1.03
2008	Equatorial G.	0.95	0.94	1.00	0.95	0.95	0.96
2009	Equatorial G.	1.01	0.96	0.95	1.01	1.01	1.01
2010	Equatorial G.	0.93	0.97	1.00	0.94	0.94	0.95
2011	Equatorial G.	0.99	0.97	1.00	0.99	0.99	0.99
2012	Equatorial G.	0.99	0.97	1.00	0.99	0.99	0.99
2013	Equatorial G.	1.05	0.97	0.94	1.04	1.04	1.04
2014	Equatorial G.	0.94	0.98	0.90	0.95	0.95	0.96
2015	Equatorial G.	0.99	1.00	1.00	0.99	0.99	0.99
2002	Eritrea						
2003	Eritrea	1.03	0.99	1.03	1.03	1.03	1.02
2004	Eritrea	1.03	0.99	1.03	1.03	1.03	1.02
2005	Eritrea	4.74	1.01	4.74	1.07	1.07	1.07
2006	Eritrea	1.00	1.00	1.00	1.00	1.00	1.00
2007	Eritrea	1.00	1.01	1.00	1.00	1.00	1.00
2008	Eritrea	1.20	1.00	1.20	1.19	1.19	1.18
2009	Eritrea	1.00	1.00	1.00	1.00	1.00	1.00
2010	Eritrea	1.00	1.00	1.00	1.00	1.00	1.00
2011	Eritrea	1.10	1.00	1.10	1.09	1.09	1.08
2012	Eritrea	1.00	0.96	1.00	1.00	1.00	1.00
2013	Eritrea	1.07	0.95	1.07	1.07	1.06	1.06
2014	Eritrea	1.05	0.97	1.05	1.05	1.05	1.05
2015	Eritrea	1.05	0.99	1.05	1.05	1.04	1.04
2002	Ethiopia						
2003	Ethiopia	1.01	1.01	1.01	1.01	1.01	1.01
2004	Ethiopia	3.22	1.01	3.22	1.22	1.22	1.22
2005	Ethiopia	1.45	1.01	1.45	1.43	1.41	1.39
2006	Ethiopia	1.74	1.01	1.74	1.71	1.69	1.66
2007	Ethiopia	1.46	1.01	1.46	1.44	1.42	1.40
2008	Ethiopia	1.27	1.01	1.27	1.25	1.24	1.23
2009	Ethiopia	1.22	1.04	1.22	1.21	1.20	1.19
2010	Ethiopia	1.16	1.01	1.16	1.15	1.15	1.14
2011	Ethiopia	1.01	1.01	1.01	1.01	1.01	1.01
2012	Ethiopia	0.13	1.19	0.13	0.28	0.38	0.45
2013	Ethiopia	1.29	0.60	1.30	1.28	1.26	1.24
2014	Ethiopia	1.10	1.01	1.10	1.09	1.09	1.09
2015	Ethiopia	1.06	1.03	1.07	1.06	1.06	1.06
2002	Ghana						
2003	Ghana	0.99	0.99	1.00	0.99	0.99	0.98
2004	Ghana	0.99	0.99	1.00	0.99	0.99	0.99
2005	Ghana	0.96	0.96	0.98	0.96	0.96	0.96
2006	Ghana	1.00	1.00	1.02	1.00	1.00	1.00

2007	Ghana	0.99	0.99	0.99	0.99	0.99	0.99
2008	Ghana	0.99	0.99	0.99	0.99	0.99	0.99
2009	Ghana	1.03	1.03	1.00	1.03	1.03	1.03
2010	Ghana	0.99	0.99	0.99	0.99	0.99	0.99
2011	Ghana	1.00	1.00	1.01	1.00	1.00	0.99
2012	Ghana	1.03	1.00	1.02	1.03	1.03	1.03
2013	Ghana	1.00	1.03	0.95	1.00	1.00	1.00
2014	Ghana	1.00	1.04	0.94	1.01	1.01	1.02
2015	Ghana	1.00	1.00	1.00	1.00	1.00	1.00
2002	Guinea						
2003	Guinea	1.07	1.01	1.07	1.06	1.06	1.06
2004	Guinea	1.06	1.02	1.06	1.06	1.05	1.05
2005	Guinea	1.05	1.06	1.02	1.05	1.05	1.05
2006	Guinea	1.01	1.01	1.03	1.01	1.01	1.01
2007	Guinea	1.00	0.99	0.98	1.00	1.00	1.00
2008	Guinea	1.06	1.02	1.00	1.06	1.06	1.06
2009	Guinea	1.09	1.01	0.98	1.08	1.08	1.08
2010	Guinea	0.96	0.96	1.00	0.96	0.96	0.96
2011	Guinea	1.00	0.99	0.99	1.00	1.00	1.00
2012	Guinea	1.08	0.97	1.00	1.08	1.07	1.07
2013	Guinea	0.96	1.07	0.94	0.96	0.96	0.96
2014	Guinea	0.99	1.04	0.92	1.00	1.00	1.00
2015	Guinea	0.96	0.97	0.98	0.96	0.96	0.96
2002	Guinea-B.						
2003	Guinea-B.	1.03	0.99	1.03	1.03	1.03	1.02
2004	Guinea-B.	1.03	0.99	1.03	1.03	1.02	1.02
2005	Guinea-B.	1.00	1.00	1.00	1.00	1.00	1.00
2006	Guinea-B.	1.40	1.00	1.40	1.40	1.40	1.40
2007	Guinea-B.	1.00	0.98	1.00	1.00	1.00	0.99
2008	Guinea-B.	1.00	0.97	1.00	1.00	0.99	0.99
2009	Guinea-B.	0.97	1.03	0.98	0.98	0.98	0.98
2010	Guinea-B.	1.00	0.99	1.00	1.00	1.00	1.00
2011	Guinea-B.	1.00	0.97	1.00	0.99	0.99	0.99
2012	Guinea-B.	1.00	1.01	1.00	1.01	1.01	1.01
2013	Guinea-B.	0.97	1.04	0.97	0.97	0.98	0.98
2014	Guinea-B.	0.97	0.97	0.96	0.97	0.97	0.97
2015	Guinea-B.	1.00	1.04	1.01	1.01	1.01	1.01
2002	Kenya						
2003	Kenya	1.01	1.01	1.01	1.01	1.01	1.01
2004	Kenya	1.14	1.01	1.21	1.14	1.14	1.14
2005	Kenya	1.07	1.00	1.10	1.07	1.07	1.07
2006	Kenya	1.17	0.98	1.22	1.17	1.17	1.16
2007	Kenya	1.22	0.98	1.25	1.21	1.20	1.19
2008	Kenya	1.18	1.02	1.19	1.18	1.17	1.16

2009	Kenya	1.15	1.01	1.14	1.14	1.14	1.13
2010	Kenya	1.14	0.99	1.13	1.13	1.13	1.12
2011	Kenya	1.14	1.04	1.12	1.14	1.13	1.13
2012	Kenya	1.03	1.00	1.04	1.03	1.03	1.03
2013	Kenya	1.03	1.00	1.02	1.03	1.03	1.03
2014	Kenya	1.06	1.01	1.04	1.06	1.05	1.05
2015	Kenya	1.09	1.02	1.06	1.08	1.08	1.08
2002	Lesotho						
2003	Lesotho	0.96	0.95	1.06	0.95	0.95	0.94
2004	Lesotho	0.98	0.97	1.06	0.98	0.98	0.98
2005	Lesotho	1.00	1.00	0.99	1.00	1.00	1.00
2006	Lesotho	1.12	0.99	1.12	1.11	1.11	1.10
2007	Lesotho	1.22	0.97	1.21	1.22	1.22	1.22
2008	Lesotho	1.14	1.00	1.16	1.13	1.13	1.12
2009	Lesotho	1.11	1.01	1.13	1.11	1.10	1.10
2010	Lesotho	1.11	1.01	1.12	1.11	1.11	1.10
2011	Lesotho	0.98	1.01	0.99	0.98	0.99	0.99
2012	Lesotho	1.03	1.01	1.02	1.03	1.03	1.03
2013	Lesotho	0.99	1.01	0.95	0.99	0.99	0.99
2014	Lesotho	0.95	1.01	0.90	0.95	0.95	0.96
2015	Lesotho	1.04	1.01	1.00	1.04	1.03	1.03
2002	Liberia						
2003	Liberia	1.00	1.00	1.00	1.00	1.00	1.00
2004	Liberia	1.02	1.00	1.02	1.02	1.02	1.02
2005	Liberia	1.00	1.00	0.99	1.00	1.00	1.00
2006	Liberia	1.83	1.01	1.83	1.83	1.83	1.83
2007	Liberia	1.00	1.00	0.98	1.00	1.00	1.00
2008	Liberia	0.75	0.77	1.00	0.77	0.78	0.79
2009	Liberia	0.85	0.92	0.96	0.85	0.86	0.87
2010	Liberia	1.00	1.00	1.00	1.00	1.00	1.00
2011	Liberia	1.00	0.98	1.00	0.99	0.99	0.99
2012	Liberia	1.00	1.00	1.00	1.00	1.00	1.00
2013	Liberia	0.96	1.01	0.96	0.96	0.96	0.97
2014	Liberia	0.92	0.98	0.93	0.93	0.93	0.93
2015	Liberia	1.00	1.01	1.00	1.00	1.00	1.00
2002	Madagascar						
2003	Madagascar	1.00	1.02	1.00	1.00	1.00	1.00
2004	Madagascar	1.00	1.01	1.00	1.00	1.00	1.00
2005	Madagascar	1.00	0.95	1.00	1.00	1.00	1.00
2006	Madagascar	1.86	0.99	1.86	1.86	1.86	1.86
2007	Madagascar	0.79	0.98	1.00	0.80	0.82	0.83
2008	Madagascar	0.71	0.98	1.00	0.73	0.74	0.76
2009	Madagascar	0.92	1.00	0.98	0.92	0.93	0.93
2010	Madagascar	1.38	1.02	1.01	1.35	1.32	1.30

2011	Madagascar	0.39	0.93	1.00	0.44	0.49	0.53
2012	Madagascar	1.00	1.00	1.00	1.00	1.00	1.00
2013	Madagascar	1.01	1.01	1.00	1.01	1.01	1.01
2014	Madagascar	1.00	1.00	1.00	1.00	1.00	1.00
2015	Madagascar	1.00	1.07	1.00	1.00	1.00	1.00
2002	Malawi						
2003	Malawi	1.01	1.01	1.01	1.01	1.01	1.01
2004	Malawi	0.93	0.93	1.00	0.93	0.94	0.94
2005	Malawi	1.01	1.01	1.05	1.01	1.01	1.01
2006	Malawi	1.01	0.95	1.05	1.01	1.01	1.01
2007	Malawi	1.05	0.97	1.08	1.05	1.05	1.05
2008	Malawi	1.08	0.96	1.11	1.08	1.08	1.07
2009	Malawi	1.11	1.00	1.07	1.11	1.10	1.10
2010	Malawi	1.11	1.03	1.06	1.11	1.10	1.10
2011	Malawi	1.09	1.01	1.04	1.09	1.09	1.08
2012	Malawi	1.11	1.01	1.08	1.10	1.10	1.10
2013	Malawi	1.04	1.03	1.05	1.04	1.04	1.04
2014	Malawi	1.06	1.02	1.03	1.06	1.06	1.06
2015	Malawi	1.06	1.02	1.04	1.06	1.05	1.05
2002	Mali						
2003	Mali	1.10	1.00	1.14	1.10	1.10	1.11
2004	Mali	1.05	0.99	1.12	1.06	1.06	1.06
2005	Mali	1.00	1.01	1.00	1.00	1.00	1.00
2006	Mali	1.10	1.00	1.12	1.10	1.10	1.10
2007	Mali	1.05	1.00	1.05	1.05	1.05	1.05
2008	Mali	1.05	1.02	1.04	1.05	1.05	1.05
2009	Mali	1.04	1.03	0.96	1.04	1.04	1.04
2010	Mali	1.05	1.03	1.01	1.05	1.05	1.05
2011	Mali	1.00	1.01	1.00	1.00	1.01	1.01
2012	Mali	1.00	0.98	1.00	1.00	1.00	1.00
2013	Mali	0.96	1.01	0.94	0.96	0.96	0.96
2014	Mali	0.93	1.00	0.87	0.93	0.93	0.94
2015	Mali	1.01	1.03	1.00	1.01	1.01	1.01
2002	Mauritania						
2003	Mauritania	1.03	0.99	1.03	1.03	1.03	1.03
2004	Mauritania	1.03	1.00	1.03	1.03	1.03	1.02
2005	Mauritania	1.34	0.99	1.34	1.34	1.34	1.34
2006	Mauritania	1.48	1.00	1.13	1.44	1.40	1.37
2007	Mauritania	0.82	0.94	1.00	0.83	0.84	0.85
2008	Mauritania	0.78	0.94	1.00	0.80	0.81	0.82
2009	Mauritania	1.14	1.03	0.98	1.13	1.12	1.12
2010	Mauritania	1.03	1.01	1.00	1.03	1.03	1.03
2011	Mauritania	0.83	0.94	1.00	0.84	0.85	0.86
2012	Mauritania	0.95	0.98	1.00	0.95	0.95	0.96

2013	Mauritania	0.81	1.00	0.97	0.82	0.83	0.84
2014	Mauritania	0.90	0.98	0.93	0.91	0.91	0.92
2015	Mauritania	1.13	1.02	1.00	1.12	1.12	1.11
2002	Morocco						
2003	Morocco	5.36	0.96	6.25	1.15	1.15	1.15
2004	Morocco	1.17	0.98	1.31	1.16	1.16	1.15
2005	Morocco	1.04	1.01	1.07	1.03	1.03	1.03
2006	Morocco	0.98	0.98	1.03	0.98	0.98	0.98
2007	Morocco	0.94	0.95	1.01	0.94	0.95	0.95
2008	Morocco	1.07	0.98	1.13	1.06	1.06	1.06
2009	Morocco	1.03	0.99	1.05	1.03	1.03	1.03
2010	Morocco	1.10	1.01	1.10	1.10	1.09	1.09
2011	Morocco	1.08	1.00	1.11	1.08	1.07	1.07
2012	Morocco	1.16	1.00	1.16	1.15	1.14	1.14
2013	Morocco	1.08	1.03	1.08	1.07	1.07	1.07
2014	Morocco	1.00	1.01	1.00	1.00	1.00	1.00
2015	Morocco	1.15	1.06	1.16	1.14	1.13	1.13
2002	Mozambique						
2003	Mozambique	0.93	0.93	0.93	0.93	0.93	0.93
2004	Mozambique	1.02	1.00	1.04	1.02	1.02	1.02
2005	Mozambique	0.97	0.97	0.98	0.97	0.97	0.97
2006	Mozambique	1.03	1.02	1.03	1.03	1.03	1.03
2007	Mozambique	1.03	1.01	1.03	1.03	1.03	1.03
2008	Mozambique	1.01	1.00	1.01	1.01	1.01	1.01
2009	Mozambique	1.01	1.01	1.01	1.01	1.01	1.01
2010	Mozambique	1.02	1.02	1.01	1.02	1.02	1.02
2011	Mozambique	1.00	1.00	1.01	1.00	1.00	1.00
2012	Mozambique	1.00	1.02	1.00	1.00	1.00	1.00
2013	Mozambique	1.02	1.00	1.02	1.02	1.02	1.02
2014	Mozambique	1.01	1.00	1.01	1.01	1.01	1.01
2015	Mozambique	1.01	1.01	1.01	1.01	1.01	1.01
2002	Namibia						
2003	Namibia	1.00	0.99	1.05	1.00	1.00	1.00
2004	Namibia	1.16	0.99	1.24	1.16	1.17	1.17
2005	Namibia	1.33	1.00	1.41	1.32	1.30	1.29
2006	Namibia	1.37	1.00	1.45	1.36	1.34	1.33
2007	Namibia	1.29	1.00	1.31	1.28	1.27	1.26
2008	Namibia	1.16	1.00	1.17	1.16	1.15	1.14
2009	Namibia	1.07	1.00	1.08	1.07	1.07	1.07
2010	Namibia	1.13	1.00	1.14	1.12	1.12	1.12
2011	Namibia	1.10	1.00	1.10	1.10	1.09	1.09
2012	Namibia	1.06	1.01	1.06	1.05	1.05	1.05
2013	Namibia	1.03	1.00	1.03	1.03	1.03	1.03
2014	Namibia	0.95	1.02	0.95	0.96	0.96	0.96

2015	Namibia	1.05	1.01	1.05	1.04	1.04	1.04
2002	Niger						
2003	Niger	0.98	1.00	1.06	0.98	0.98	0.98
2004	Niger	1.03	0.99	1.03	1.03	1.02	1.02
2005	Niger	1.28	0.94	1.28	1.28	1.28	1.28
2006	Niger	1.23	1.00	1.23	1.24	1.24	1.24
2007	Niger	1.19	1.00	1.21	1.19	1.18	1.18
2008	Niger	0.85	0.98	1.00	0.86	0.87	0.88
2009	Niger	1.40	1.02	1.37	1.38	1.37	1.35
2010	Niger	1.57	1.04	1.39	1.51	1.47	1.42
2011	Niger	1.17	0.99	1.17	1.16	1.15	1.14
2012	Niger	1.13	1.00	1.13	1.13	1.12	1.12
2013	Niger	0.89	1.02	0.89	0.90	0.91	0.92
2014	Niger	0.92	1.01	0.90	0.92	0.93	0.93
2015	Niger	0.69	0.97	0.69	0.72	0.74	0.77
2002	Nigeria						
2003	Nigeria	1.13	0.64	2.03	1.16	1.19	1.21
2004	Nigeria	1.36	0.97	1.57	1.34	1.33	1.32
2005	Nigeria	1.12	0.93	1.32	1.12	1.12	1.12
2006	Nigeria	1.12	0.98	1.43	1.12	1.12	1.12
2007	Nigeria	0.98	0.88	1.02	0.98	0.99	0.99
2008	Nigeria	1.09	0.92	1.28	1.09	1.09	1.09
2009	Nigeria	1.12	1.12	1.14	1.12	1.11	1.10
2010	Nigeria	1.00	0.95	1.07	1.00	1.00	1.00
2011	Nigeria	1.01	0.97	1.04	1.01	1.01	1.01
2012	Nigeria	1.03	0.95	1.04	1.03	1.03	1.03
2013	Nigeria	1.03	0.98	1.11	1.03	1.03	1.03
2014	Nigeria	1.03	1.00	1.07	1.03	1.03	1.03
2015	Nigeria	1.08	1.08	1.06	1.08	1.07	1.07
2002	Rwanda						
2003	Rwanda	0.96	0.93	1.02	0.96	0.96	0.96
2004	Rwanda	1.06	1.02	1.09	1.06	1.06	1.06
2005	Rwanda	1.33	0.99	1.40	1.33	1.32	1.31
2006	Rwanda	1.33	1.01	1.37	1.31	1.30	1.28
2007	Rwanda	1.23	0.92	1.24	1.22	1.21	1.20
2008	Rwanda	1.18	1.02	1.19	1.18	1.17	1.16
2009	Rwanda	1.08	1.02	1.08	1.08	1.08	1.07
2010	Rwanda	1.09	1.02	1.09	1.09	1.09	1.08
2011	Rwanda	1.07	1.01	1.07	1.07	1.06	1.06
2012	Rwanda	1.08	1.01	1.08	1.08	1.07	1.07
2013	Rwanda	1.04	1.01	1.04	1.04	1.04	1.04
2014	Rwanda	0.99	1.01	0.99	0.99	0.99	1.00
2015	Rwanda	1.05	1.01	1.05	1.05	1.05	1.04
2002	Senegal						

2003	Senegal	1.03	0.98	1.04	1.03	1.02	1.02
2004	Senegal	1.03	0.99	1.05	1.02	1.02	1.02
2005	Senegal	1.01	1.03	1.00	1.01	1.01	1.01
2006	Senegal	1.02	1.00	1.04	1.02	1.02	1.02
2007	Senegal	1.29	1.01	1.32	1.29	1.29	1.29
2008	Senegal	1.04	0.98	1.10	1.04	1.03	1.03
2009	Senegal	1.09	1.03	1.09	1.08	1.08	1.08
2010	Senegal	1.08	1.02	1.08	1.07	1.07	1.07
2011	Senegal	1.05	1.00	1.04	1.05	1.05	1.05
2012	Senegal	1.08	0.99	1.01	1.07	1.07	1.07
2013	Senegal	0.99	1.04	0.98	0.99	1.00	1.00
2014	Senegal	1.00	1.02	0.95	1.00	1.00	1.00
2015	Senegal	1.15	1.06	1.02	1.15	1.14	1.13
2002	Sierra Leone						
2003	Sierra Leone	1.06	1.03	1.06	1.06	1.06	1.06
2004	Sierra Leone	1.06	1.01	1.06	1.05	1.05	1.05
2005	Sierra Leone	1.00	1.00	1.00	1.00	1.00	1.00
2006	Sierra Leone	1.05	1.02	1.05	1.05	1.05	1.05
2007	Sierra Leone	1.00	1.01	1.00	1.00	1.00	1.00
2008	Sierra Leone	1.00	1.01	1.00	1.00	1.00	1.00
2009	Sierra Leone	0.95	1.01	0.95	0.95	0.95	0.96
2010	Sierra Leone	1.00	1.02	1.00	1.00	1.00	1.00
2011	Sierra Leone	1.00	1.01	1.00	1.00	1.00	1.00
2012	Sierra Leone	1.00	1.00	1.00	1.00	1.00	1.00
2013	Sierra Leone	0.94	1.01	0.94	0.95	0.95	0.95
2014	Sierra Leone	0.88	1.01	0.88	0.89	0.89	0.90
2015	Sierra Leone	1.00	1.01	1.00	1.00	1.00	1.00
2002	Sudan						
2003	Sudan	0.69	0.98	1.04	0.71	0.73	0.75
2004	Sudan	0.86	0.98	1.05	0.87	0.88	0.89
2005	Sudan	0.92	0.98	1.00	0.92	0.93	0.93
2006	Sudan	0.96	0.96	1.05	0.96	0.96	0.96
2007	Sudan	0.99	0.95	1.00	0.99	0.99	0.99
2008	Sudan	1.00	1.01	1.00	1.00	1.00	1.00
2009	Sudan	0.95	1.00	0.95	0.95	0.95	0.96
2010	Sudan	1.00	1.01	1.00	1.00	1.00	1.00
2011	Sudan	1.00	0.99	1.00	1.00	1.00	1.00
2012	Sudan	1.05	0.98	1.00	1.05	1.04	1.04
2013	Sudan	0.94	1.04	0.94	0.94	0.94	0.95
2014	Sudan	0.95	1.02	0.90	0.95	0.95	0.96
2015	Sudan	0.99	0.97	1.00	0.99	0.99	0.99
2002	Togo						
2003	Togo	1.04	0.99	1.05	1.04	1.04	1.04
2004	Togo	1.03	0.99	1.03	1.03	1.03	1.03

2005	Togo	1.03	1.00	1.03	1.03	1.03	1.03
2006	Togo	1.04	0.79	1.04	1.04	1.04	1.04
2007	Togo	0.80	1.00	0.99	0.81	0.82	0.84
2008	Togo	0.85	0.84	1.00	0.86	0.86	0.87
2009	Togo	0.98	0.98	0.95	0.98	0.98	0.98
2010	Togo	0.99	0.97	1.00	0.99	0.99	0.99
2011	Togo	0.99	0.97	1.00	0.99	0.99	0.99
2012	Togo	1.00	0.98	1.00	1.00	1.00	1.00
2013	Togo	0.96	1.01	0.95	0.96	0.96	0.96
2014	Togo	0.93	1.00	0.91	0.93	0.94	0.94
2015	Togo	1.02	1.03	1.00	1.02	1.02	1.02
2002	Uganda						
2003	Uganda	1.09	1.03	1.09	1.09	1.09	1.08
2004	Uganda	1.70	0.99	1.70	1.07	1.07	1.07
2005	Uganda	1.34	1.00	1.34	1.32	1.31	1.30
2006	Uganda	0.99	1.01	0.99	0.99	0.99	0.99
2007	Uganda	1.09	1.01	1.09	1.09	1.09	1.08
2008	Uganda	1.12	1.01	1.11	1.11	1.11	1.11
2009	Uganda	1.03	1.02	1.03	1.03	1.03	1.03
2010	Uganda	0.99	0.97	0.99	0.99	0.99	0.99
2011	Uganda	1.03	1.03	1.03	1.03	1.03	1.03
2012	Uganda	1.02	1.01	1.02	1.02	1.02	1.02
2013	Uganda	1.04	1.02	1.04	1.04	1.04	1.04
2014	Uganda	1.04	1.00	1.03	1.04	1.04	1.04
2015	Uganda	1.05	1.01	1.04	1.05	1.05	1.05
2002	Zambia						
2003	Zambia	1.26	1.01	1.18	1.26	1.25	1.24
2004	Zambia	1.03	1.01	1.04	1.03	1.03	1.03
2005	Zambia	1.21	1.00	1.20	1.21	1.20	1.20
2006	Zambia	1.19	1.04	1.16	1.19	1.18	1.18
2007	Zambia	1.45	1.02	1.44	1.43	1.42	1.40
2008	Zambia	1.23	1.00	1.24	1.22	1.21	1.20
2009	Zambia	1.13	1.03	1.14	1.13	1.12	1.12
2010	Zambia	1.11	1.01	1.13	1.10	1.10	1.09
2011	Zambia	1.11	1.02	1.14	1.10	1.10	1.10
2012	Zambia	1.07	1.01	1.07	1.06	1.06	1.05
2013	Zambia	1.06	1.02	1.05	1.06	1.06	1.06
2014	Zambia	1.04	1.01	1.03	1.04	1.04	1.04
2015	Zambia	1.10	1.01	1.13	1.09	1.09	1.08

Table S6. Calculation Data Q3-2017-Q4-2018 for 8 NGO.

Period	NGO	Amount invested	Reached total individuals	Reached total families	Water inf. provided
Q3-2017	Action Contre la Faim	$6,931,108	21,690	4,338	-
Q3-2017	International Organization for Migration	$19,790,673	1,016,015	191,918	28
Q3-2017	Oxfam	$1,847,941	19,350	8,701	-
Q3-2017	Save the Children	$1,855,369	53,140	10,628	-
Q3-2017	Solidarités International	$1,968,133	32,350	9,568	-
Q3-2017	UN Women	$435,870	1,420	7,084	-
Q3-2017	United Nations High Commission for Refugees	$2,842,363	14,348	2,648	-
Q3-2017	World Vision International	$306,560	10,044	2,009	-
Q4-2017	Action Contre la Faim	$6,931,108	24,430	5,886	-
Q4-2017	International Organization for Migration	$19,790,673	2,784,681	745,844	93
Q4-2017	Oxfam	$1,847,941	244,837	26,134	1,824
Q4-2017	Save the Children	$1,855,369	379,526	90,065	-
Q4-2017	Solidarités International	$1,968,133	342,644	84,482	-
Q4-2017	UN Women	$435,870	4,288	1,597	-
Q4-2017	United Nations High Commission for Refugees	$2,842,363	56,390	29,686	-
Q4-2017	World Vision International	$306,560	42,845	9,749	-
Q1-2018	Action Contre la Faim	$107,362	21,610	4,322	-
Q1-2018	International Organization for Migration	$23,108,409	1,310,138	643,991	1,708
Q1-2018	Oxfam	$292,740	331,994	77,720	1,582
Q1-2018	Save the Children	$1,804,557	22,175	7,108	-
Q1-2018	Solidarités International	$442,666	56,591	6,492	-

Q1-2018	UN Women	$340,040	2,345	1,295	-
Q1-2018	United Nations High Commission for Refugees	$38,855,611	8,250	3,815	-
Q1-2018	World Vision International	$2,033,989	-	3,500	-
Q2-2018	Action Contre la Faim	$107,362	7,590	1,790	-
Q2-2018	International Organization for Migration	$23,108,409	475,198	151,352	709
Q2-2018	Oxfam	$292,740	1,078	4,528	-
Q2-2018	Save the Children	$1,804,557	1,875	1,298	-
Q2-2018	Solidarités International	$442,666	105,185	18,237	-
Q2-2018	UN Women	$340,040	31,046	2,762	-
Q2-2018	United Nations High Commission for Refugees	$38,855,611	119,440	24,006	-
Q2-2018	World Vision International	$2,033,989	48,880	9,832	-
Q3-2018	Action Contre la Faim	$107,362	17,630	3,582	-
Q3-2018	International Organization for Migration	$23,108,409	232,651	60,590	644
Q3-2018	Oxfam	$292,740	202,896	11,717	-
Q3-2018	Save the Children	$1,804,557	21,700	13,917	-
Q3-2018	Solidarités International	$442,666	16,700	50	-
Q3-2018	UN Women	$340,040	6,000	1,200	-
Q3-2018	United Nations High Commission for Refugees	$38,855,611	30,968	11,718	-
Q3-2018	World Vision International	$2,033,989	9,965	1,585	-
Q4-2018	Action Contre la Faim	$107,362	23,980	4,828	-
Q4-2018	International Organization for Migration	$23,108,409	57,693	12,381	664
Q4-2018	Oxfam	$292,740	46,382	9,066	-
Q4-2018	Save the Children	$1,804,557	9,300	2,400	-
Q4-2018	Solidarités International	$442,666	109,000	33,018	-

Q4-2018	UN Women	$340,040	32,790	6,558	-
Q4-2018	United Nations High Commission for Refugees	$38,855,611	1,699	0	-
Q4-2018	World Vision International	$2,033,989	3,780	756	-

Table S7. Results for input oriented DEA calculations of NGO model.

Period	NGO	I	II	III	IV
Q3-2017	Action Contre la Faim	0.06	0.08	0.06	0.08
Q3-2017	International Organization for Migration	1.00	1.00	1.00	1.00
Q3-2017	Oxfam	0.33	0.33	0.33	0.33
Q3-2017	Save the Children	0.57	0.62	0.57	0.62
Q3-2017	Solidarités International	0.40	0.42	0.40	0.42
Q3-2017	UN Women	1.00	1.00	1.00	1.00
Q3-2017	United Nations High Commission for Refugees	0.10	0.14	0.10	0.14
Q3-2017	World Vision International	0.66	1.00	0.66	1.00
Q4-2017	Action Contre la Faim	0.02	0.04	0.02	0.04
Q4-2017	International Organization for Migration	0.78	1.00	0.78	1.00
Q4-2017	Oxfam	1.00	1.00	1.00	1.00
Q4-2017	Save the Children	1.00	1.00	1.00	1.00
Q4-2017	Solidarités International	0.88	0.89	0.88	0.89
Q4-2017	UN Women	0.60	0.83	0.60	0.83
Q4-2017	United Nations High Commission for Refugees	0.22	0.24	0.22	0.24
Q4-2017	World Vision International	0.68	1.00	0.68	1.00
Q1-2018	Action Contre la Faim	0.18	1.00	0.18	1.00
Q1-2018	International Organization for Migration	0.11	1.00	0.11	1.00

Q1-2018	Oxfam	1.00	1.00	1.00	1.00
Q1-2018	Save the Children	0.02	0.06	0.02	0.06
Q1-2018	Solidarités International	0.11	0.29	0.11	0.29
Q1-2018	UN Women	0.14	0.38	0.14	0.38
Q1-2018	United Nations High Commission for Refugees	-	0.00	-	0.00
Q1-2018	World Vision International	0.01	0.05	0.01	0.05
Q2-2018	Action Contre la Faim	0.41	1.00	0.41	1.00
Q2-2018	International Organization for Migration	1.00	1.00	1.00	1.00
Q2-2018	Oxfam	0.38	0.56	0.38	0.56
Q2-2018	Save the Children	0.02	0.06	0.02	0.06
Q2-2018	Solidarités International	1.00	1.00	1.00	1.00
Q2-2018	UN Women	0.38	0.55	0.38	0.55
Q2-2018	United Nations High Commission for Refugees	0.02	0.04	0.02	0.04
Q2-2018	World Vision International	0.12	0.13	0.12	0.13
Q3-2018	Action Contre la Faim	0.83	1.00	0.83	1.00
Q3-2018	International Organization for Migration	1.00	1.00	1.00	1.00
Q3-2018	Oxfam	1.00	1.00	1.00	1.00
Q3-2018	Save the Children	0.19	0.73	0.19	0.73
Q3-2018	Solidarités International	0.05	0.24	0.05	0.24
Q3-2018	UN Women	0.09	0.32	0.09	0.32
Q3-2018	United Nations High Commission for Refugees	0.01	0.01	0.01	0.01
Q3-2018	World Vision International	0.02	0.05	0.02	0.05
Q4-2018	Action Contre la Faim	0.91	1.00	0.91	1.00
Q4-2018	International Organization for Migration	1.00	1.00	1.00	1.00

Q4-2018	Oxfam	0.64	0.67	0.64	0.67
Q4-2018	Save the Children	0.02	0.06	0.02	0.06
Q4-2018	Solidarités International	1.00	1.00	1.00	1.00
Q4-2018	UN Women	0.39	0.42	0.39	0.42
Q4-2018	United Nations High Commission for Refugees	-	0.00	-	0.00
Q4-2018	World Vision International	0.01	0.05	0.01	0.05

Table S8. Results output-oriented DEA calculations NGO model.

Period	NGO	V	VI	VII	VIII
Q3-2017	Action Contre la Faim	0.06	0.06	0.06	0.06
Q3-2017	International Organization for Migration	1.00	1.00	1.00	1.00
Q3-2017	Oxfam	0.33	0.42	0.33	0.42
Q3-2017	Save the Children	0.57	0.60	0.57	0.60
Q3-2017	Solidarités International	0.40	0.44	0.40	0.44
Q3-2017	UN Women	1.00	1.00	1.00	1.00
Q3-2017	United Nations High Commission for Refugees	0.10	0.10	0.10	0.10
Q3-2017	World Vision International	0.66	1.00	0.66	1.00
Q4-2017	Action Contre la Faim	0.02	0.02	0.02	0.02
Q4-2017	International Organization for Migration	0.78	1.00	0.78	1.00
Q4-2017	Oxfam	1.00	1.00	0.65	0.65
Q4-2017	Save the Children	1.00	1.00	1.00	1.00
Q4-2017	Solidarités International	0.88	0.90	0.88	0.90
Q4-2017	UN Women	0.60	0.77	0.60	0.77
Q4-2017	United Nations High Commission for Refugees	0.22	0.24	0.22	0.24

Q4-2017	World Vision International	0.68	1.00	0.68	1.00
Q1-2018	Action Contre la Faim	0.18	1.00	0.18	1.00
Q1-2018	International Organization for Migration	0.11	1.00	0.11	1.00
Q1-2018	Oxfam	1.00	1.00	1.00	1.00
Q1-2018	Save the Children	0.02	0.06	0.02	0.06
Q1-2018	Solidarités International	0.11	0.17	0.11	0.17
Q1-2018	UN Women	0.14	0.16	0.14	0.16
Q1-2018	United Nations High Commission for Refugees	-	0.01	-	0.01
Q1-2018	World Vision International	0.01	0.03	0.01	0.03
Q2-2018	Action Contre la Faim	0.41	1.00	0.41	1.00
Q2-2018	International Organization for Migration	1.00	1.00	1.00	1.00
Q2-2018	Oxfam	0.38	0.42	0.38	0.42
Q2-2018	Save the Children	0.02	0.05	0.02	0.05
Q2-2018	Solidarités International	1.00	1.00	1.00	1.00
Q2-2018	UN Women	0.38	0.41	0.38	0.41
Q2-2018	United Nations High Commission for Refugees	0.02	0.25	0.02	0.25
Q2-2018	World Vision International	0.12	0.37	0.12	0.37
Q3-2018	Action Contre la Faim	0.83	1.00	0.83	1.00
Q3-2018	International Organization for Migration	1.00	1.00	1.00	1.00
Q3-2018	Oxfam	1.00	1.00	1.00	1.00
Q3-2018	Save the Children	0.19	0.93	0.19	0.93
Q3-2018	Solidarités International	0.05	0.08	0.05	0.08
Q3-2018	UN Women	0.09	0.10	0.09	0.10
Q3-2018	United Nations High Commission for Refugees	0.01	0.19	0.01	0.19

Q3-2018	World Vision International	0.02	0.10	0.02	0.10
Q4-2018	Action Contre la Faim	0.91	1.00	0.91	1.00
Q4-2018	International Organization for Migration	1.00	1.00	1.00	1.00
Q4-2018	Oxfam	0.64	0.65	0.64	0.65
Q4-2018	Save the Children	0.02	0.09	0.02	0.09
Q4-2018	Solidarités International	1.00	1.00	1.00	1.00
Q4-2018	UN Women	0.39	0.40	0.39	0.40
Q4-2018	United Nations High Commission for Refugees	-	0.02	-	0.02
Q4-2018	World Vision International	0.01	0.04	0.01	0.04

Table S9. Results for DEA bootstrap calculations with 1,000 iterations.

NPO/NGO	Period	BCC	Bias corrected	Lower bound	Upper bound
Q3-2017	Action Contre la Faim	0.41	0.30	0.21	0.57
Q4-2017	Action Contre la Faim	0.07	0.04	0.03	0.09
Q1-2018	Action Contre la Faim	1.00	1.00	1.00	1.00
Q2-2018	Action Contre la Faim	1.00	1.00	1.00	1.00
Q3-2018	Action Contre la Faim	1.00	1.00	1.00	1.00
Q4-2018	Action Contre la Faim	1.00	1.00	1.00	1.00
Q3-2017	Oxfam	0.82	0.66	0.55	0.90
Q4-2017	Oxfam	0.65	0.42	0.21	0.85
Q1-2018	Oxfam	1.00	1.00	1.00	1.00
Q2-2018	Oxfam	0.42	0.48	0.29	0.95
Q3-2018	Oxfam	1.00	1.00	1.00	1.00
Q4-2018	Oxfam	0.65	0.62	0.51	0.82
Q3-2017	Save the Children	1.00	0.92	0.88	1.00
Q4-2017	Save the Children	1.00	0.95	0.03	1.00
Q1-2018	Save the Children	0.09	0.04	0.00	0.11
Q2-2018	Save the Children	0.07	0.05	0.04	0.11
Q3-2018	Save the Children	1.00	1.00	1.00	1.00
Q4-2018	Save the Children	0.09	0.06	0.00	0.11
Q3-2017	Solidarités International	0.90	0.72	0.58	1.00
Q4-2017	Solidarités International	0.94	0.53	0.14	1.00
Q1-2018	Solidarités International	0.17	0.05	0.00	0.45
Q2-2018	Solidarités International	1.00	1.00	1.00	1.00

Q3-2018	Solidarités International	0.08	0.06	0.00	0.28
Q4-2018	Solidarités International	1.00	1.00	1.00	1.00
Q3-2017	UN Women	1.00	0.50	0.05	1.00
Q4-2017	UN Women	0.77	0.47	0.20	1.00
Q1-2018	UN Women	0.16	0.05	0.00	0.41
Q2-2018	UN Women	0.41	0.47	0.32	1.00
Q3-2018	UN Women	0.10	0.07	0.00	0.29
Q4-2018	UN Women	0.40	0.38	0.34	0.76
Q3-2017	World Vision International	1.00	0.49	0.03	1.00
Q4-2017	World Vision International	1.00	1.00	1.00	1.00
Q1-2018	World Vision International	0.05	0.01	0.00	0.05
Q2-2018	World Vision International	0.54	0.56	0.50	1.00
Q3-2018	World Vision International	0.12	0.09	0.00	0.29
Q4-2018	World Vision International	0.04	0.00	0.00	0.06

Table S10. Results for DEA window analysis, window width (w) =2 and w = 3, integrating O_3.

**w = 2**	**Q3-2017**	**Q4-2017**	**Q1-2018**	**Q2-2018**	**Q3-2018**	**Q4-2018**	**Mean**
	0.02	0.02					
		0.02	1.00				
ACF			1.00	1.00			0.71
				1.00	1.00		
					1.00	1.00	
	0.37	1.00					
		1.00	1.00				
IOM			1.00	0.42			0.87
				1.00	0.91		
					1.00	1.00	
	0.10	1.00					
		1.00	1.00				
Oxfam			1.00	0.06			0.70
				0.39	1.00		
					1.00	0.48	
	0.14	1.00					
		0.72	0.06				
Save the Childr.			0.06	0.01			0.30
				0.05	0.53		
					0.40	0.07	
SI	0.10	0.90					0.44
		0.63	0.16				

**w = 3**	**Q3-2017**	**Q4-2017**	**Q1-2018**	**Q2-2018**	**Q3-2018**	**Q4-2018**	**Mean**
	0.02	0.02	1.00				
ACF		0.02	1.00	1.00			0.76
			1.00	1.00	1.00		
				1.00	1.00	1.00	
	0.37	1.00	1.00				
IOM		1.00	1.00	0.41			0.78
			1.00	0.42	0.38		
				1.00	0.91	0.94	
	0.07	1.00	1.00				
Oxfam		1.00	1.00	0.06			0.62
			1.00	0.06	0.61		
				0.22	1.00	0.48	
	0.10	0.72	0.06				
Save the Childr.		0.72	0.06	0.01			0.19
			0.06	0.01	0.12		
				0.03	0.35	0.06	
	0.07	0.63	0.16				
SI		0.63	0.16	0.30			0.36
			0.17	0.31	0.05		
				0.72	0.08	1.00	
UN Woman	0.09	0.15	0.16				0.13
		0.15	0.16	0.09			

			0.17	0.31			
				1.00	0.08		
					0.08	1.00	
	0.43	0.77					
		0.15	0.16				
UN Women			0.16	0.09			0.25
				0.20	0.09		
					0.05	0.29	
	0.03	0.24					
		0.18	0.01				
UNHCR							0.11
				0.25	0.08		
					0.19	0.01	
	0.23	1.00					
		0.13	0.03				
WVI			0.03	0.12			0.20
				0.36	0.06		
					0.06	0.03	

			0.16	0.09	0.02		
				0.17	0.05	0.29	
	0.02	0.18	0.01				
		0.18	0.01	0.04			
UNHCR							0.07
			0.01	0.09	0.02		
				0.25	0.08	0.00	
	0.03	0.13	0.03				
		0.13	0.03	0.09			
WVI							0.08
			0.03	0.12	0.03		
				0.29	0.05	0.02	

Results for DEA window analysis, window width (w) =2 and w = 3, excluding O_3

**w = 2**	**Q3-2017**	**Q4-2017**	**Q1-2018**	**Q2-2018**	**Q3-2018**	**Q4-2018**	**Mean**
	0.06	0.07					
		0.07	1.00				
ACF			0.18	0.06			0.47
				0.50	1.00		
					0.74	1.00	
	0.10	0.65					
		0.65	1.00				
Oxfam			1.00	0.06			0.63
				0.39	1.00		
					1.00	0.48	
	0.14	1.00					
		1.00	0.08				
Save the Childr.			0.02	0.00			0.36
				0.07	0.76		
					0.42	0.08	
	0.11	0.94					
		0.94	0.17				
SI			0.11	0.21			0.46
				1.00	0.08		
					0.08	1.00	
UN Women	0.43	0.77					0.25
		0.26	0.16				

**w = 3**	**Q3-2017**	**Q4-2017**	**Q1-2018**	**Q2-2018**	**Q3-2018**	**Q4-2018**	**Mean**
	0.06	0.07	1.00				
ACF		0.07	1.00	0.41			0.58
			1.00	0.41	0.83		
				0.37	0.74	1.00	
	0.10	0.65	1.00				
Oxfam		0.65	1.00	0.06			0.57
			1.00	0.06	0.61		
				0.22	1.00	0.48	
	0.14	1.00	0.08				
Save the Childr.		1.00	0.08	0.01			0.26
			0.09	0.02	0.18		
				0.04	0.41	0.08	
	0.11	0.94	0.17				
SI		0.94	0.17	0.31			0.41
			0.17	0.32	0.05		
				0.73	0.08	1.00	
	0.15	0.26	0.16				
UN Woman		0.26	0.16	0.09			0.16
			0.16	0.09	0.02		
				0.17	0.05	0.29	
WVI	0.10	0.44	0.04				0.15
		0.44	0.04	0.13			

			0.14	0.08			
				0.20	0.09		
					0.05	0.29	
	0.23	1.00					
		0.44	0.04				
WVI			0.01	0.02			0.25
				0.54	0.09		
					0.07	0.03	

	0.05	0.15	0.03	
		0.36	0.07	0.03
